# Sperm-borne miR-216b modulates cell proliferation during early embryo development via *K-RAS*

**DOI:** 10.1038/s41598-019-46775-8

**Published:** 2019-07-17

**Authors:** Maíra Bianchi Rodrigues Alves, Rubens Paes de Arruda, Tiago Henrique Camara De Bem, Shirley Andrea Florez-Rodriguez, Manoel Francisco de Sá Filho, Clémence Belleannée, Flávio Vieira Meirelles, Juliano Coelho da Silveira, Felipe Perecin, Eneiva Carla Carvalho Celeghini

**Affiliations:** 1 Department of Animal Reproduction, School of Veterinary Medicine and Animal Science, Universidade de São Paulo, Pirassununga, São Paulo Brazil; 2 Department of Veterinary Medicine, School of Animal Science and Food Engineering, Universidade de São Paulo, Pirassununga, São Paulo Brazil; 3 Alta Genetics of Brazil, Uberaba, Minas Gerais Brazil; 4 Department of Obstetrics, Gynecology and Reproduction, Université Laval, Quebec, Quebec, Canada

**Keywords:** Embryology, Development, Gene expression, miRNAs

## Abstract

Semen fertilizing potential is dependent upon the morphological, functional and molecular attributes of sperm. Sperm microRNAs (miRNAs) were recently shown to hold promise regarding their association with different fertility phenotypes. However, their role in fertility regulation remains to be determined. We postulated that sperm miRNAs might regulate early embryonic development. From this perspective, sperm quality and 380 sperm miRNAs were investigated in frozen–thawed semen from high (HF; 54.3 ± 1.0% pregnancy rate) and low (LF; 41.5 ± 2.3%) fertility bulls. Out of nine miRNAs that showed different levels in sperm cells, miR-216b was present at lower levels in HF sperm cells and zygotes. Among miR-216b target genes (*K-RAS*, *BECN1* and *JUN*), *K-RAS*, related to cell proliferation, revealed a higher level in HF two-cell embryos. First cleavage rate, blastocyst cell number and division number were also higher in HF. In addition, by using a model based on polyspermy embryos, we demonstrated an increase in miR-216b levels in zygotes associated with sperm cell entry. Our results shed light on a possible mechanism of paternal contribution involving sperm-borne miR-216b that modulates levels of miR-216b in zygotes and *K-RAS* in two-cell embryos. This modulation might regulate early development by interfering with the first cleavage and blastocyst quality.

## Introduction

All sperm attributes, including morphological, functional and molecular characteristics, are important for male fertility^1,2^. As a result of the complexity of these attributes, recording pregnancy rates is the most satisfactory procedure that allows the assessment of semen fertilization potential; however, this is expensive and laborious^3^. Thus, an investigation into the efficiency of each sperm attribute as a predictor of male fertility is essential to further our understanding of their relative contributions. In this context, sperm cells should have the ability to: (1) reach the fertilization site; (2) fertilize the ovum; and (3) contribute to early embryonic development^2^. Sperm morphological and functional aspects, referred to herein as sperm quality attributes (SQA), are directly related to the sperm’s ability to reach the oviduct and fertilize the ovum. Sperm motility, abnormalities, membrane integrity, mitochondria function and DNA status are examples of SQA that often show high correlation with male fertility. As such, SQA are frequently used to assess the fertility potential of frozen–thawed semen batches^1,4–6^. However, since male fertility potential is dependent upon several sperm characteristics^7^, restricting evaluation to these features might be insufficient to predict fertility for some semen samples^8^. Molecular sperm signatures are frequently linked to their contribution to early embryonic development, which involves delivery of the following to the embryo: sperm DNA, mRNAs, proteins and non-coding RNAs such as microRNAs (miRNAs) that appear to be related to healthy sperm and male fertility potential^2,9,10^.

MicroRNAs are small non-coding RNA molecules (~22 nt) that show a high degree of conservation among species and are involved in modulation of protein translation^11^. The canonical biogenesis pathway consists of RNA polymerase II-mediated transcription of primary miRNA transcripts (pri-miRNA; ~200 nt) from DNA, which are cleaved by Drosha and DGCR8 to produce precursor miRNAs (pre-miRNA; ~70 nt). Pre-miRNA is transported from the nucleus to the cytoplasm by Exportin 5, where it undergoes cleavage by Dicer and RNA-binding protein TRBP to form a miRNA duplex of ~22 nt. Only one strand binds to a complex of Argonaute proteins to form the RISC complex (RNA-induced silencing complex) whose activity is dependent on binding of the miRNA to the 3′ untranslated region (3′ UTR) of its target gene: perfect binding induces degradation of the target mRNA; while imperfect binding prevents translation of the target mRNA^11^. MiRNAs are currently known for their roles in spermatogenesis^12^, sperm maturation^13^ and embryo development^14^. Human sperm cell miRNAs were first described by Ostermeier *et al*. (2005)^15,16^ and exhibit different profiles according to fertility status^17,18^. Specific “sperm-borne” miRNAs such as miR-34c and miR-449b are important for the first cleavage in mouse and bovine embryos, respectively^10,19,20^.

Based on (1) the dependence on morphological, functional and molecular sperm attributes for male fertility success, and, (2) the potential of sperm-borne miRNAs to play a role in fertility by regulating embryo development, we hypothesized that initial steps of embryo development are under the control of specific miRNAs delivered by sperm cells. Thus, the aims of this study were to: (1) determine the SQA and miRNA profiles of semen samples from high and low fertility bulls; (2) investigate the relative levels of sperm miRNAs and target genes in embryos from high and low fertility sperm samples; (3) investigate how alterations in miRNAs could regulate embryo development; and (4) demonstrate that sperm cells are capable of delivering miRNAs to the zygote.

## Results

### Experiment 1: Lower level of miR-216b in sperm cells and zygotes is associated with a high level of *K-RAS* in two-cell embryos and an increase in first cleavage rate and blastocyst cell number

#### HF and LF semen samples display similarity regarding sperm quality attributes

Sperm quality characterization was first performed on three commercial semen batches from each bull to give a total of 18 batches. The results showed that the majority of the evaluated characteristics were similar for the two different groups of samples— refer to Supplementary Table S1. Selection of the best semen batch from each bull was performed according to Supplementary Table S2 to give a total of three batches from HF bulls (High Fertility; n = 3) and three batches from LF bulls (Low Fertility; n = 3). In that regard, the following formula was used:

$$(1\times {\rm{P}}{\rm{R}}{\rm{O}}{\rm{G}})+[3\times (100-{\rm{M}}{\rm{A}}{\rm{J}})]+[2\times (100-{\rm{M}}{\rm{I}}{\rm{N}})]+(3\times {\rm{P}}{\rm{I}}{\rm{A}}{\rm{I}}{\rm{H}}),$$

where PROG was progressive motility, MAJ was major defects, MIN was minor defects and PIAIH was sperm with plasma and acrosome membrane integrity and high mitochondrial membrane potential. The individual characteristics of each batch are described in Supplementary Tables S3 and S4. The morphological and functional analyses of the selected batches showed that sperm cells from HF and LF bulls were similar with regard to sperm motility, abnormalities, kinetic, plasma and acrosome membrane integrity, mitochondrial function, production of ROS, lipid peroxidation and DNA fragmentation according to data presented in Tables 1 and 2.

**Table 1:** Mean and SEM of sperm quality attributes (sperm motility, concentration, abnormalities and kinetic) evaluated in the selected commercial semen batches.

Sperm quality analyses of the selected batches	Fertility groups	
	HF^a^ (n = 3)	LF^b^ (n = 3)
***Sperm motility***, ***concentration and abnormalities***		
Straw volume (mL)	0.25	0.25
Subjective motility (%)	68.3 ± 0.8	60.8 ± 3.6
Vigor (1–5)	3.5 ± 0.1	3.3 ± 0.2
Sperm concentration (×10^6^ sperm/mL)	90.0 ± 11.3	88.3 ± 11.6
Major defects (%)	16.5 ± 3.9	24.3 ± 1.5
Minor defects (%)	6.5 ± 1.8	4.3 ± 0.8
Total defects (%)	23.0 ± 5.7	28.6 ± 2.3
Total of sperm/straw (×10^6^)	22.5 ± 2.8	22.1 ± 2.9
Total of motility sperm/straw (×10^6^)	15.3 ± 1.9	13.5 ± 2.4
***Sperm kinetic***		
Total motility (%)	82.2 ± 1.4	73.9 ± 5.3
Progressive motility (%)	57.2 ± 5.7	58.5 ± 4.4
Rapid cells (%)	75.6 ± 0.2	69.3 ± 4.6
Curvilinear velocity (VCL; µm/s)	144.7 ± 8.0	172.0 ± 10.7
Progressive velocity (VSL; µm/s)	75.7 ± 6.7	87.4 ± 3.4
Path velocity (VAP; µm/s)	95.0 ± 4.3	104.7 ± 5.6
Linearity (LIN; %)	52.5 ± 4.9	51.0 ± 2.1
Straightness (STR; %)	79.3 ± 4.2	83.5 ± 1.3
Wobble (WOB; %)	65.9 ± 2.7	61.1 ± 2.1
Lateral head displacement (ALH; µm)	2.6 ± 0.1	3.3 ± 0.3
Beat cross frequency (BCF; Hz)	21.8 ± 0.7	24.2 ± 1.1

Differences were considered significant when P < 0.05. ^a^High fertility; ^b^Low fertility. SEM: standard error of the mean.

**Table 2:** Mean and SEM of sperm quality attributes (sperm membranes integrity and function, production of ROS, lipid peroxidation and DNA fragmentation) evaluated in the selected commercial semen batches.

Sperm quality analyses of the selected batches	Fertility groups	
	HF^a^ (n = 3)	LF^b^ (n = 3)
***Sperm membranes integrity***, ***mitochondrial membrane function and ROS production by fluorescence microscopy***		
PIAIH^c^ (%)	48.9 ± 4.7	45.2 ± 6.1
Plasma membrane integrity (%)	51.5 ± 5.1	48.5 ± 6.4
Acrosome membrane integrity (%)	60.7 ± 3.6	59.4 ± 4.1
High mitochondrial membrane potential (%)	62.6 ± 7.9	54.7 ± 7.2
ROS^d^ (%)	5.2 ± 0.9	10.7 ± 4.9
***Sperm membranes integrity***, ***mitochondrial membrane function***, ***ROS production***, ***lipid peroxidation and DNA fragmentation by flow cytometry***		
Plasma membrane integrity (%)	49.5 ± 0.3	45.6 ± 8.2
Acrosome membrane integrity (%)	83.9 ± 2.3	78.3 ± 3.7
Plasma and acrosome membrane integrity (%)	49.3 ± 0.3	45.4 ± 8.3
Plasma membrane integrity and high mitochondrial membrane potential (%)	24.4 ± 2.1	26.3 ± 2.9
Plasma membrane integrity and mitochondrial positive ROS (%)	5.3 ± 1.6	4.1 ± 1.0
Mitochondrial positive ROS (ua)	621.0 ± 76.2	678.3 ± 23.5
Lipid peroxidation (ua)	2,502.0 ± 395.6	2,764.9 ± 921.8
DNA fragmentation (%)	5.8 ± 0.1	5.7 ± 0.1

Differences were considered significant when P < 0.05. ^a^High fertility; ^b^Low fertility; ^c^Sperm with plasma and acrosome membrane integrity and high mitochondrial potential; ^d^Production of ROS (reactive oxygen species). SEM: standard error of the mean.

#### Nine miRNAs are present at a different relative level between sperm cells from HF and LF

**Figure 1:**
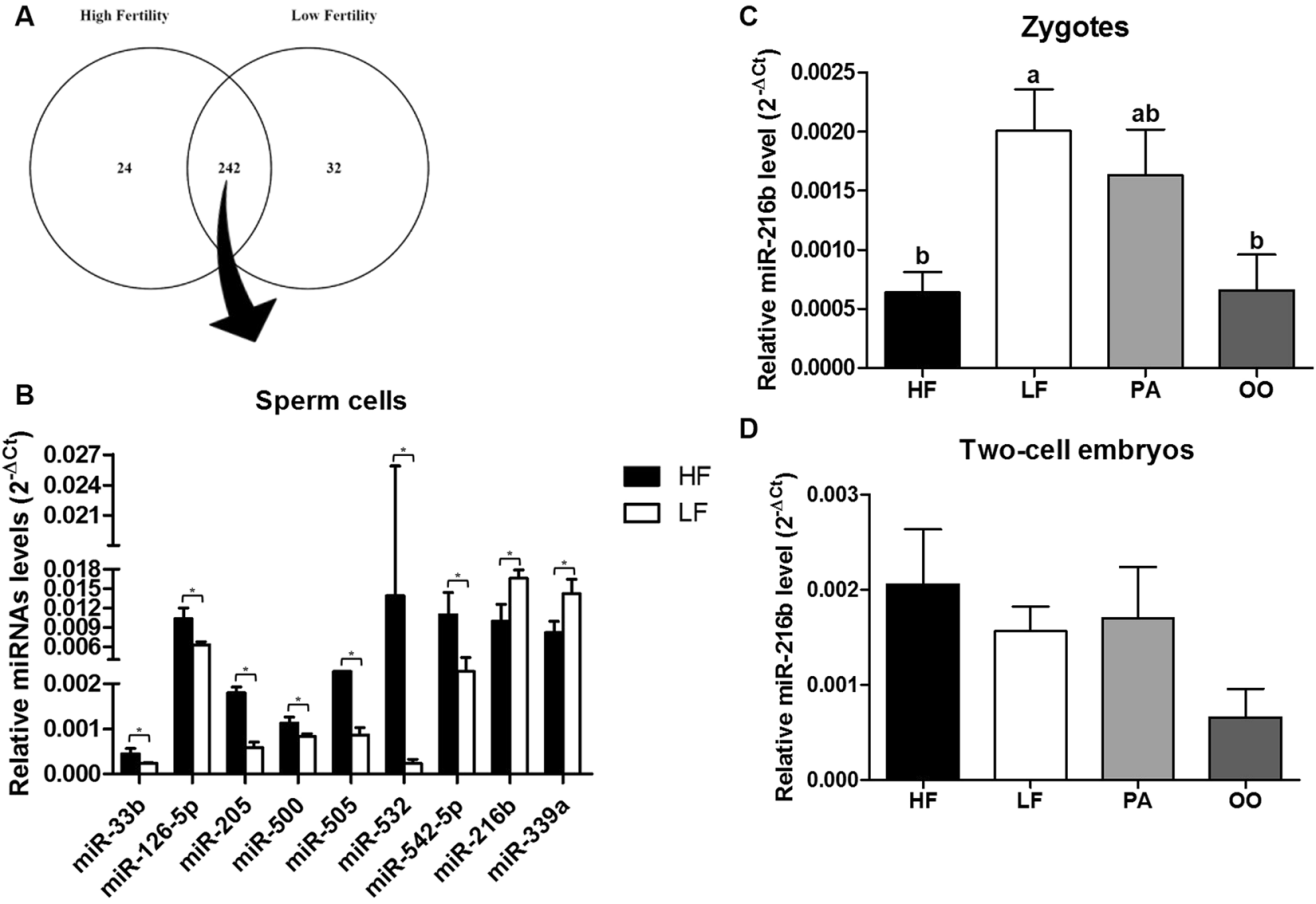
MiRNA analysis in sperm cells and in *in vitro*-produced embryos. (**A**) Venn diagram of 298 detected miRNAs in sperm cells from high fertility (HF) or low fertility (LF) bulls from a profile of 380 miRNAs. (**B**) Relative levels of the differentially abundant miRNAs (P < 0.10) between sperm cells from high fertility and low fertility bulls. (**C**) MiR-216b relative level in zygotes from high fertility (HF), low fertility (LF), parthenogenetic (PA) and mature oocyte (OO) groups. (**D**) miR-216b relative level in two-cell embryos from high fertility (HF), low fertility (LF), parthenogenetic (PA) and mature oocyte (OO) groups. Asterisks (*) indicate difference between the groups with a significance level of P < 0.10. ^a,b^Different letters indicate statistical difference (P < 0.05) between groups. All quantitative data are presented as means and SEM.

A panel of 380 miRNAs was evaluated in sperm cells from selected batches. Among these, 24 miRNAs were exclusively detected in sperm cells from HF bulls, 32 miRNAs were exclusively detected in sperm cells from LF bulls and 242 miRNAs were detected in both groups, as shown in Supplementary Table S5 and Fig. 1A. Nine out of 242 miRNAs showed different relative levels between HF and LF sperm samples with a P value of 0.10 (Fig. 1B). Different relative levels of bta-miR-205, -505 and -532 showed a statistical significance of P < 0.05; bta-miR-33b, -126-5p, -216b, -339a, -500 and -542-5p presented a P value between 0.05 and 0.10. Bta-miR-205, -505, -532, -33b, -126-5p, -500 and -542-5p were found in higher abundance in sperm cells from HF bulls, whereas bta-miR-216b and -339a were found in higher abundance in sperm cells from LF bulls (Fig. 1B).

#### Higher first cleavage rate is detected in HF embryos

After evaluation of sperm quality and miRNA profile, sperm from selected HF and LF semen batches (characteristics are described in Tables 1 and 2**)** were used to produce IVF embryos; parthenogenetic embryos were also produced and used as controls. In each replicate of embryo production, pools of 20 to 25 oocytes were previously divided into drops intended specifically for evaluation and collection of zygotes, two-cell embryos and blastocysts at a specific time interval. Fertilized zygotes were classified and collected according the rate of second polar body extrusion evaluated 16 to 18 hours post insemination (hpi); this rate was found to be similar between HF and LF zygotes as shown in Table 3. However, the first cleavage rate, evaluated 28 to 30 hpi in HF and LF, and 20 to 22 hours post activation in parthenogenetic embryos, was higher in embryos from HF bulls and parthenogenesis than in embryos from LF bulls (Table 3). The cleavage and blastocyst rates were evaluated from the same group of oocytes/embryos. The cleavage rate assessed on day-4 of development were similar among the three groups. Blastocyst rate evaluated on day-7 showed a higher percentage in the parthenogenetic embryo group compared with HF and LF, as shown in Table 3.

**Table 3:** Pre-implantation development rates of bovine embryos produced *in vitro*.

Fertility Groups	2^nd^ PB^a^			1^st^ cleavage^b^			Cleavage and blastocysts^c^				
							Oocytes	Cleavage		Blastocysts	
	Oocytes	N	Mean ± SEM	Oocytes	N	Mean ± SEM		N	Mean ± SEM	N	Mean ± SEM
HF^d^	329	160	48.6 ± 2.1	273	75	27.5 ± 3.8^a^	335	251	74.9 ± 3.6	111	33.1 ± 2.9^b^
LF^e^	322	139	43.2 ± 4.2	278	52	18.7 ± 3.7^b^	375	290	77.3 ± 2.9	115	30.7 ± 4.1^b^
PA^f^	—	—	—	227	70	30.8 ± 3.7^a^	300	228	76.0 ± 2.9	126	42.0 ± 2.9^a^

^a^Second polar body extrusion (PB; evaluated 16 to 18 hpi), ^b^first cleavage (evaluated 28 to 30 hpi or 20 to 22 hpa), ^c^cleavage on day-4 and blastocysts on day-7 were evaluated in embryos produced *in vitro* using high and low fertility semen samples and embryos produced *in vitro* by parthenogenesis. ^a,b^Different letters on the same column indicate statistical difference (P < 0.05) between groups. ^d^High fertility; ^e^Low fertility; ^f^Parthenogenetic. Hpi: hours post insemination. Hpa: hours post activation. Mean and SEM are presented as percentages. SEM: standard error of the mean.

#### Lower level of miR-216b and higher level of its target gene *K-RAS* are detected in HF zygotes and two-cell embryos, respectively

**Figure 2:**
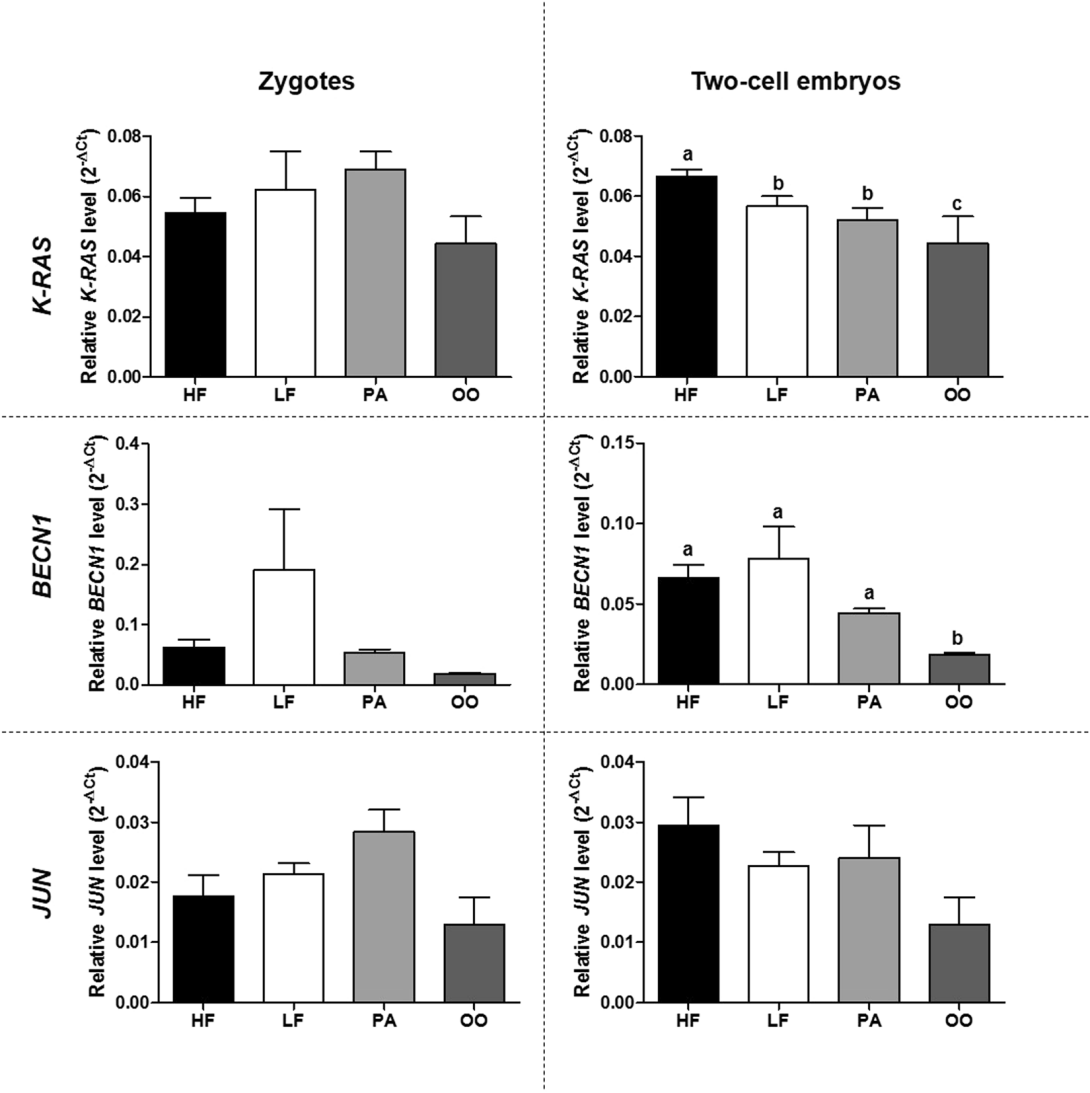
Analyses of miR-216b target genes in mature oocytes and in *in vitro*-produced embryos. *K-RAS*, *BECN1* and *JUN* relative level in zygotes and two-cell embryos from high fertility (HF), low fertility (LF), parthenogenetic (PA) and mature oocyte (OO) groups. ^a,b,c^Different letters indicate statistical difference (P < 0.05) between groups. All quantitative data are presented as means and SEM.

The relative levels of nine miRNAs (Fig. 1B) that were different between HF and LF sperm samples were investigated in zygotes and two-cell embryos produced from HF and LF bulls. Parthenogenetic embryos and mature oocytes were used as controls for this analysis. Among the miRNAs evaluated, it was observed that the relative level of miR-216b was lower in HF zygotes and oocytes (Fig. 1C). In two-cell embryos, none of the miRNAs, including miR-216b, showed a difference in relative levels between HF and LF embryos (Fig. 1D). The relative levels of the nine miRNAs investigated in embryos are listed in Supplementary Tables S6 and S7. After establishing that miR-216b presented the same differential pattern in both sperm cells and zygotes from HF and LF groups, the relative levels of miR-216b target genes were investigated in embryos. In order to determine miR-216b target genes, we first performed a homology analysis of bta-miR-216b with hsa-miR-216b-5p, which showed that the miRNAs shared the same nucleotide sequence (aaaucucugcaggcaaauguga), revealing 100% homology. Target genes of hsa-miR-216b were then investigated and selected according to two criteria: (1) strong validated mRNA-miR216b interaction (according to the miRTarBase platform); and (2) genes that play a relevant role in embryo development. Thus, *K-RAS*, *BECN1* and *JUN* target genes were selected^21–24^. Furthermore, the 3′ UTR was conserved between *K-RAS*, *BECN1* and *JUN* sequences from humans and cattle. The target gene levels were investigated in mature oocytes as well as in zygotes and two-cell embryos from the HF, LF and parthenogenetic groups as shown in Fig. 2. Relative levels of *K-RAS*, *BECN1* and *JUN* showed no difference between zygotes and oocytes. However, the *K-RAS* gene was present at a higher relative level in two-cell embryos from the HF group as shown in Fig. 2. The *BECN1* gene was present at a lower relative level in mature oocytes, but showed the same relative level in embryos from the HF and LF groups.

#### HF embryos display a higher cell number in blastocysts and a higher number of cell divisions up to the day-7 blastocyst stage

**Figure 3:**
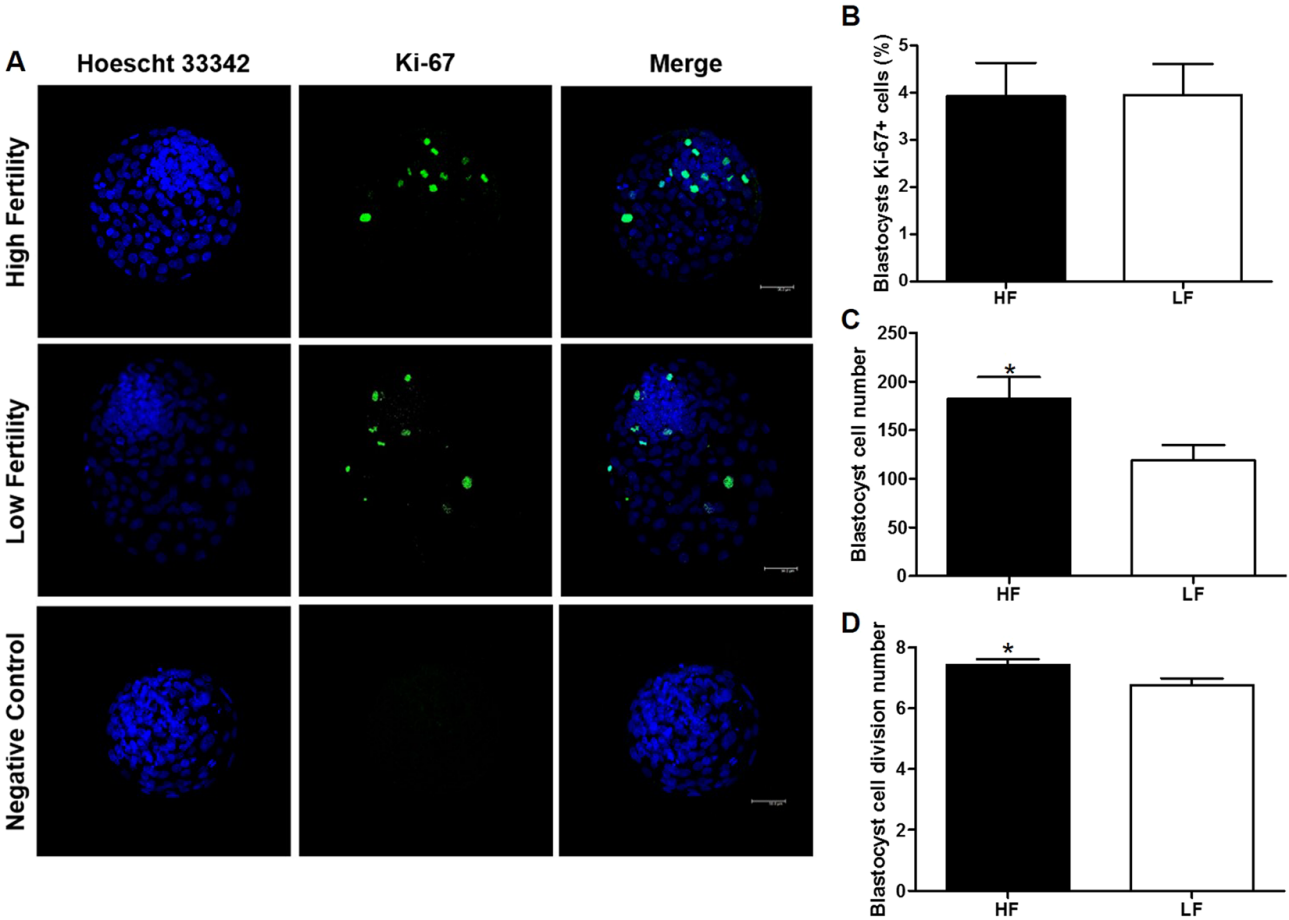
Blastocyst qualitative analysis. (**A**) Confocal representative photomicrographs of Ki-67 immunostaining in blastocysts from high fertility and low fertility groups and negative control. Scale bar: 50 μm. (**B**) Percentage of cells in high fertility (HF) and low fertility (LF) blastocysts that were marked with Ki-67 antibody. (**C**) Cell numbers in high fertility (HF) and low fertility (LF) blastocysts stained with Hoechst 33342. (**D**) Cell division number in high fertility (HF) and low fertility (LF) blastocysts. Asterisks (*) indicate difference between the groups with a significance level of P < 0.05. All quantitative data are presented as means and SEM.

Since *K-RAS* gene expression is associated with cell proliferation, embryo quality was investigated with respect to blastocyst proliferation, diameter, stage and blastocyst cell number as well the number of cell divisions up to the day-7 blastocyst stage. Immunostaining was performed with Ki-67 to investigate the proliferation rate of blastocyst embryos. This revealed that HF and LF blastocysts proliferated at a similar rate (Fig. 3A,B). In a similar manner, the diameter of blastocysts from HF (235.4 ± 18.3 µm) and LF (218.4 ± 16.7 µm) groups were similar. Moreover, the percentage of early blastocysts (HF: 3.3 ± 2.0%; LF: 6.8 ± 3.5%), blastocysts (HF: 28.3 ± 5.1%; LF: 25.2 ± 5.7%), expanded blastocysts (HF: 51.7 ± 5.9%; LF: 43.7 ± 3.9%) and hatched blastocysts (HF: 16.7 ± 5.7%; LF: 24.3 ± 5.1%) showed no difference between the groups. However, the cell number and number of cell divisions were higher in HF blastocysts than in LF blastocysts (Fig. 3C,D). In HF blastocysts the number of cells and the number of cell divisions were 182.4 ± 22.4 and 7.4 ± 0.2, respectively; in LF blastocysts they were 119.3 ± 15.5 and 6.8 ± 0.2, respectively. Furthermore, developmental kinetic parameters were investigated in embryos produced by IVF performed for 8 hours, instead of the 18 hours performed previously, in order to investigate the possibility of a fertilization delay with LF sperm cells. Although cleavage rates for four-cell embryos (HF: 25.4 ± 4.2%; LF: 26.8 ± 3.2%) were similar in this study, HF (35.1 ± 6.8%) showed a tendency (P = 0.07) to produce a higher percentage of two-cell embryos than LF (28.5 ± 6.7%) as shown in Supplementary Table S8.

### Experiment 2: Higher level of miR-216b is associated with polyspermic zygotes

#### Sperm concentration of 8 × 10^6^ sperm/mL per IVF drop is efficient at increasing the polyspermic zygote rate

**Figure 4:**
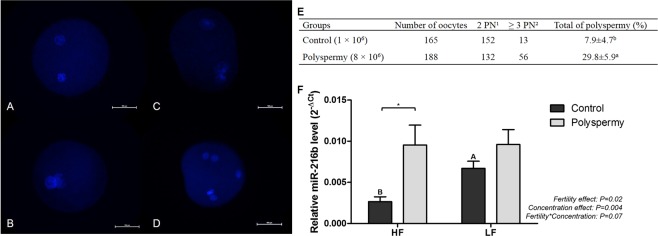
Polyspermic embryo validation and analysis of miR-216b relative level. (**A**–**D**) Representative fluorescence microscopy photomicrographs of pronuclei from zygotes of control and polyspermic groups. Scale bar: 100 μm. In (***A***,***B***), zygotes with two pronuclei. In (***C***,***D***), zygotes with more than three pronuclei. (**E**) Evaluation of pronucleus (PN) and polyspermy rate in bovine zygotes produced *in vitro* with control IVF (sperm concentration of 1 × 10^6^ sperm/mL) or polyspermy IVF (sperm concentration of 8 × 10^6^ sperm/mL). ^1^2 PN: two pronuclei; ^2^ ≥ 3 PN: more than three pronuclei. (**F**) MiR-216b relative level in zygotes produced *in vitro* with control IVF (sperm concentration of 1 × 10^6^ sperm/mL) or polyspermy IVF (sperm concentration of 8 × 10^6^ sperm/mL) using semen samples from high (HF) and low (LF) fertility bulls. Capital letters indicate difference (P < 0.05) between groups (HF *vs*. LF) with the same treatment (Control). Asterisk (*) indicates difference (P < 0.05) between the treatments (control *vs*. polyspermy) in the same group. All quantitative data are presented as means and SEM.

In order to investigate whether sperm cells are able to deliver miR-216b to zygotes, we proposed a new model based on polyspermic embryos. Our intent with this model was to show that by increasing the number of sperm cells per oocyte, the level of sperm-borne miR-216b in zygotes might be increased. With this aim, we first conducted a study to determine the sperm concentration that could increase polyspermic zygote formation. Previous studies conducted in our lab have shown that a sperm concentration of 8 × 10^6^ sperm/mL per IVF drop results in enhanced induction of polyspermy in a physiological and consistent manner compared with 4 × 10^6^ sperm/mL and 16 × 10^6^ sperm/mL (see Supplementary Fig. S1**)**. Thus, to verify polyspermy induction rates, a sperm concentration of 1 × 10^6^ sperm/mL per IVF drop was considered the control and a concentration of 8 × 10^6^ sperm/mL was considered polyspermy induced. Control and polyspermy induced sperm concentrations were evaluated with regard to the rate of polyspermic zygote formation (Fig. 4A–D). The percentage of polyspermic embryos was increased 3.8 times by the polyspermy induced sperm concentration as shown in Fig. 4E. The polyspermy rate was 7.9 ± 4.7% for the control group and 29.8 ± 5.9% for the polyspermy induced group. The percentage of polyspermic zygotes presenting with exactly three pronuclei in the polyspermy group (17.5 ± 4.6%) was 2.9 times more than in the control group (6.1 ± 4.7%). In addition, the rate of polyspermic zygotes presenting four or more pronuclei was markedly lower in the control group (1.8 ± 0.4%), while the polyspermy induced group was 6.8 times higher (12.2 ± 3.1%).

#### MiR-216b is present at a higher level in polyspermic embryos

Since our data suggest that the levels of miR-216b might be modulated by sperm cells in zygotes, we expected that polyspermic embryos would have a higher level of miR-216b. Thus, after verifying polyspermy induction, the relative level of miR-216b was investigated in polyspermic and control embryos produced with selected HF and LF semen samples. The results showed that polyspermy increased the miR-216b relative level (Sperm concentration effect: P = 0.004) as shown in Fig. 4F. Furthermore, comparison between controls revealed that the relative level of miR-216b was higher in the LF group than in the HF group as shown previously.

## Discussion

Fertility success depends on many factors including female, male and environmental influences. Among the male factors, the presence of healthy sperm cells is crucial for fertility^2^. Sperm quality attributes (SQA; morphological and functional attributes) and sperm molecular aspects are directly related to healthy sperm determination. Sperm quality attributes are usually highly correlated with male fertilization potential and are frequently a useful predictor of the latter^4,25,26^. However, some semen samples exhibit satisfactory SQA coupled with unexpected low fertility^27^. Thus, sperm molecular characteristics have been studied in order to better understand these instances. A particular sperm molecular characteristic that has been described concerns sperm miRNAs. Indeed, miRNAs are important for spermatogenesis^12,28^, sperm maturation^13,29,30^ and also embryo development^10,14,20^. Furthermore, we now know, in addition to delivering DNA to embryos, sperm cells are also able to deliver RNAs and miRNAs^2,10,13,20,31,32^. Therefore, in the present study, we investigated the SQA from semen samples obtained from HF and LF bulls as well the effect of sperm miRNAs contribution to pre-implantation embryonic development. Of interest is that differences between SQA in semen samples from HF and LF might not explain the difference in fertility rates. In addition, we demonstrated that the miR-216b level in zygotes is related with the level in sperm, and that this might be related to the transcriptional changes in the relative *K-RAS* gene levels found in two-cell embryos. Moreover, the reduced level of the *K-RAS* gene in LF two-cell embryos could be associated with the reduction in LF first cleavage rate. Of note is that while blastocyst rates were unaffected, we detected a lower number of cells in blastocysts derived from the LF group suggesting a reduction in the number of cell cycles up to the day-7 blastocyst stage. In addition, we observed that polyspermy zygotes displayed a higher level of miR-216b. Thus, our results provide, for the first time, a probable regulation of early embryonic development by sperm-borne miR-216b and reveal the modulation of its levels by producing polyspermic embryos.

Different fertility phenotypes have already been associated with specific sperm miRNA profiles in cattle^18,33^ and humans^17,34^. In the present study, we used commercial frozen semen samples from bulls that showed different pregnancy rates in field trials and similar sperm morphological and functional characteristics. Although the blastocyst rate for *in vitro* embryo production was similar between the HF and LF groups according to our previous results, a parallel prospective study was performed using the same batches used in the present study and revealed a difference of ~13% on the pregnancy rate between HF and LF semen samples^35^. The goal of this prospective study was to improve the use of low fertility semen samples in fixed-time artificial insemination (FTAI) programs. In this regard, 522 suckled cows from three different herds were subjected to FTAI. It was hypothesized that the delay in time of artificial insemination in FTAI would improve pregnancy rates from LF semen samples, but this was not confirmed. Hence, the results showed that pregnancy rates were not impacted by FTAI protocol modifications. In addition, our results revealed that blastocyst rates were similar between the fertility groups. Thus, we hypothesize that differences in fertility rates were probably due to functional characteristics related to early embryo development that could involve, among other factors, molecular components/miRNAs delivered by HF and LF sperm cells.

In the present study, we detected nine miRNAs that showed a difference in their relative levels of expression between LF and HF with a statistical significance of 10%. Of interest is that a large number of other miRNAs showed differences in relative abundance between high and low fertility bulls *e*.*g*. miR-19b-3p, -27a-5p, -34c-3p, -148b-3p, -320a -502-5p and -1249^18^. Investigation of the profiles of nine sperm miRNAs revealed that one miRNA (miR-216b) was present at a higher relative level in sperm cells and also in zygotes from LF compared with HF. However, HF and LF two-cell embryos showed similar relative levels of this miRNA. Although hsa-miR-216b-5p is described in the SpermBase platform (http://spermbase.org/), to the best of our knowledge, our study is the first to report the importance of miR-216b to male fertility and early embryo development. However, in different types of cancer tumors, the role of miR-216b in inhibiting cell growth and proliferation is well described^36,37^.

MiR-216b has a limited number of target genes that have been conclusively identified. *K-RAS*, *BECN1* and *JUN* were selected from among these for the present study due to their importance in embryo development^21–24^. Only *K-RAS* showed high relative levels in HF two-cell embryos compared with LF embryos. *K-RAS* is a member of the *ras* proto-oncogene family and plays a role in cell proliferation and differentiation^38^. It is the only member of the *ras* family with an important role in embryo development^22,23^. Depletion of *K-RAS* in mice results in embryo developmental deficiency and increased embryo mortality^22,23^. However, there is a lack of information regarding the importance of *K-RAS* to embryo development in cattle. Of interest is that the 3′ UTR of *K-RAS* is completely conserved among cattle, humans and mice. Moreover, it is well described that miR-216b targets *K-RAS* in many cell types. It has been documented in pancreatic^39,40^, nasopharyngeal^41^ and renal tumors^42^ that decreasing the abundance of the *K-RAS* gene consequently leads to low cellular proliferation and differentiation. Thus, we propose that lower levels of *K-RAS* in LF two-cell embryos is a temporal effect resulting from the higher level of miR-216b in LF zygotes provided by LF sperm cells. In fact, we showed here that sperm cells from LF bulls exhibited a higher level of miR-216b than sperm cells from HF bulls. Furthermore, we showed that the miR-216b level was higher when more than one sperm cell fertilized the oocyte to produce polyspermic embryos.

Since *K-RAS* is related to cell proliferation, we investigated the rates of first cleavage, cleavage on day-4, blastocyst stage on day-7, blastocyst proliferation, blastocyst cell number and number of cell divisions during initial development. We also evaluated the two-cell and four-cell rates using a developmental kinetic model. We hypothesized that HF embryos would show more proliferation. Indeed, with respect to these parameters, first cleavage rate, blastocyst cell number and blastocyst cell division number were higher in HF embryos compared with LF. Of interest is that HF embryos underwent an additional cell cycle compared with LF embryos (see Fig. 3D). The higher cell number and the higher number of cell divisions in HF blastocysts could indicate some beneficial effect to the establishment of pregnancy. In humans, a higher number of cells in transferred embryos is highly associated with pregnancy success^43^. In fact, blastocysts are able to produce and secrete factors, called embryotropins^44^, involved in modulating endometrium transcripts mediated by interferon-tau, prostaglandin metabolism and aquaporin channels^45^. In light of this, we propose that embryos with a high first cleavage rate and high cell number could release an increased quantity of embryotropins^44^ that are then able to modulate and signal the presence of the embryo to the endometrium^45^, thereby influencing *in vivo* fertility success. However, this hypothesis is yet to be confirmed and further studies are necessary to confirm this association.

In addition to the importance of miR-216b to embryo development, we showed, using a polyspermy induction model, that we can modulate the miR-216b level in zygotes by increasing the sperm cell concentration per IVF drop. Indeed, we revealed that the increasing sperm cell entry into oocytes at fertilization is able to increase the levels of miR-216b in zygotes. We produced polyspermic zygotes by increasing the number of sperm cells per IVF drop while maintaining the oocyte physiology as intact as possible to verify that the alteration was caused by increasing the number of sperm cells entering each oocyte. The zygotes were evaluated in terms of the relative level of miR-216b. In polyspermic embryos, miR-216b showed increased relative levels, demonstrating that sperm cells might modulate miR-216b levels in the embryo. However, the relative levels of miR-216b in HF and LF polyspermy embryos were unexpectedly similar. This might be attributed to the fact that we were unable to sort the polyspermic embryos prior to PCR, and we were also unable to control the number of pronuclei generated in these embryos. In addition, the miR-216b level might be modulated by sperm-related factors that stimulate miR-216b transcription in LF embryos or that inhibit it in HF embryos; this hypothesis could justify the similar levels of miR-216b in HF and LF polyspermy embryos since the transcription process might reach a maximum rate. However, further studies are necessary to verify this probable new mechanism of sperm-borne modulation.

**Figure 5:**
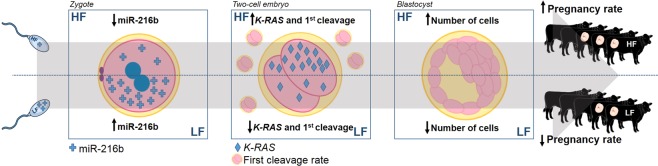
Hypothetical schematic model. Schematic figure demonstrating that sperm cells from high fertility (HF) bulls might deliver a lower level of miR-216b to zygotes than sperm from low fertility (LF) bulls. Thus, zygotes from HF may display a lower miR-216b level than zygotes from LF and its target gene, *K-RAS*, may exhibit a higher level in two-cell embryos from HF than LF. Furthermore, this might result in a higher first cleavage rate in HF embryos. These changes are probably reflected in the higher HF blastocyst cell number, which might be beneficial to *in vivo* development and thereby could increase pregnancy rates in cattle.

In conclusion, our results shed light on a probable mechanism by which sperm cells are able to contribute miR-216b to zygotes, thereby modulating early embryo development. In summary, our data showed that miR-216b was present at a lower level in sperm cells from HF bulls. This was associated with a lower relative level of miR-216b in HF zygotes and with a higher relative level of its target gene, *K-RAS*, in HF two-cell embryos, implicating an increase in first cleavage rate and blastocyst cell number (Fig. 5). Finally, our data provide a better understanding of how paternal contributions could modulate early embryo development. The present study may open new avenues for the implications of sperm miRNAs in fertility regulation and establishment of healthy pregnancy in cattle.

## Methods

### Ethics statement

All procedures performed during this study were conducted in accordance with ethical guidelines of research and animal care and were approved by the Ethics Committee on Animal Use of the School of Veterinary Medicine and Animal Science of the University of São Paulo (CEUA/FMVZ) under protocol number 1912050516. We adopted the International Guiding Principles for Biomedical Research Involving Animals (Society for the Study of Reproduction) as well. All schematic images were made by the authors or obtained from the Mind the Graph platform (https://mindthegraph.com/).

### Reagents and chemicals

Unless otherwise stated, all reagents and chemicals used were purchased from Sigma-Aldrich (St. Louis, MO, USA). Fluorescent probes used were: Hoechst 33342 (Hoechst; reference number H1399), 5,5′,6,6′-tetrachloro-1,1′,3,3′-tetraethylbenzimidazolcarbocyanine iodide (JC-1; T3168), CellROX^TM^ Deep Red (CellROX Red; C10422), SYTO™ 59 Red (Syto59; S11341), MitoSOX^TM^ Red (MitoSOX, M36008), YO-PRO^TM^-1 iodide (YoPro, Y3603), C11-BODIPY^TM^ 581/591 (Bodipy, D3861) and Acridine Orange (AO; A1301) purchased from Thermo Fisher Scientific (Waltham, Massachusetts, USA); and propidium iodide (PI; P4170), fluorescein isothiocyanate-conjugated *Pisum sativum* agglutinin (FITC-PSA; L0770) and fluorescein isothiocyanate-conjugated *Arachis hypogaea* (FITC-PNA, L7381) purchased from Sigma-Aldrich.

### Experiment 1: Lower level of miR-216b in sperm cells and zygotes is associated with a high level of *K-RAS* in two-cell embryos and an increase in first cleavage rate and blastocyst cell number

#### Experimental design

Six commercial batches of frozen–thawed semen from high fertility (HF; n = 3; 54.3 ± 1% pregnancy rate) and low fertility (LF; n = 3; 41.5 ± 2.3%) Aberdeen Angus (*Bos taurus*) bulls were used. The bulls’ field fertility rates were different (P = 0.007) and determined by the Concept Plus^®^ program developed by Alta Genetics^®^ (Uberaba, MG, Brazil). High and low fertility bulls were evaluated respectively in 396 herds and 52,259 services and in 22 herds and 3,405 services. First, all batch samples were evaluated for sperm quality. Then, one batch from each bull was selected, analysed for sperm quality and for a profile of 380 miRNAs, and then used for *in vitro* embryo production.

#### Sperm quality assessment and semen batch selection

All sperm quality analyses were conducted in duplicate by the same technician. Two semen straws (0.25 mL) from the same batch were thawed (37 °C/30 seconds) and the total volume (~500 µL) was placed in a pre-warmed tube and homogenized before proceeding with sperm quality assessment. Semen evaluation was performed as soon the semen was thawed and homogenized.

#### Assessment of sperm motility, concentration and abnormalities

Contrast phase microscopy (100× magnification) was used to evaluate (Nikon, 80i model, Tokyo, Japan) subjective sperm motility (%) and vigor (1 to 5, whereby 1 corresponds to immotile sperm cells and 5 corresponds to sperm cells with progressive and fast movement). Sperm concentration was evaluated by diluting semen in formaldehyde 4% (1:100) using a Neubauer chamber under magnification at 400×. The percentage of abnormalities was evaluated by fixing the semen sample in formaldehyde 4% and classifying 200 sperm cells as having major, minor and total defects (major plus minor)^46^ (Supplementary Table S9) using differential interference contrast microscopy (Nikon, 80i model, Tokyo, Japan) under 1,000× magnification.

#### Assessment of sperm kinetic

Sperm kinetic parameters were evaluated by Computer-Assisted Sperm Analyser (CASA) using Sperm Class Analyser (SCA, Microptics, Barcelona, Spain) software. Set up was adjusted for bovine sperm (Supplementary Table S10). The sperm concentration was adjusted to 10 × 10^6^ sperm/mL in Tyrode’s albumin lactate pyruvate medium (TALP sperm: 4.2 mg/mL sodium chloride, 1.87 mg/mL potassium chloride, 2.1 mg/mL sodium bicarbonate, 50 µg/mL sodium phosphate, 290 µg/mL calcium chloride monohydrate, 80 µg/mL magnesium chloride hexahydrate and 6.5 mg/mL Hepes, supplemented with 10 mg/mL bovine serum albumin - pH adjusted to 7.4 with NaOH 1N). Then, 10 µL of diluted semen was placed on a Makler^®^ chamber (Selfi-Medical Instruments, Haifa, Israel) and evaluated for total motility (%), progressive motility (%), rapid cells (%), curvilinear velocity (VCL, μm/s), path velocity (VAP, μm/s), progressive velocity (VSL, μm/s), linearity (LIN, %), straightness (STR, %), wobble (WOB, %), lateral head displacement (ALH, μm) and beat cross frequency (BCF, Hz).

#### Assessment of sperm membranes and reactive oxygen species production

Epifluorescence microscopy (Nikon, model 80i, Tokyo, Japan) at 1,000× magnification using a triple filter (D/F/R, C58420) with UV-2E/C (excitation 340–380 nm/emission 435–485 nm), B-2E/C (excitation 465–495 nm/emission 515–555 nm) and G-2E/C (excitation 540–525 nm/emission 605–655 nm) was used for analyses of plasma and acrosome membrane integrity and mitochondrial membrane potential, and reactive oxygen species (ROS) production. For assessment of sperm plasma and acrosome membrane integrity and mitochondrial membrane potential^47^, 2 µL of Hoechst 0.5 mg/mL, 2 µL of PI 0.5 mg/mL, 6 µL of JC-1 153 µM and 20 µL of FITC-PSA 100 µg/mL were added to 150 µL of semen diluted to 10 × 10^6^ sperm/mL in TALP sperm. The incubation was performed at 37 °C/8 minutes. Two hundred sperm cells were then classified (percentage) according to PIAIH (sperm cells with plasma and acrosome membrane integrity, and high potential of mitochondrial membrane), integrity of plasma membrane, integrity of acrosome membrane and high potential of mitochondrial membrane. Production of ROS^48^ was evaluated by the addition of 1.2 µL of CellROX Red 2.5 mM and 2 µL of Hoechst 0.5 mg/mL to 150 µL diluted semen (10 × 10^6^ sperm/mL). The incubation was performed at 37 °C/30 minutes. Samples were then centrifuged at 600 *g* for 5 minutes and resuspended in TALP sperm. Two hundred sperm cells were then classified (percentage) as either positive or negative for ROS production.

#### Semen batch selection

The selection of one semen batch per bull was performed according to the highest value obtained using the formula:

$$(1\times {\rm{P}}{\rm{R}}{\rm{O}}{\rm{G}})+[3\times (100-{\rm{M}}{\rm{A}}{\rm{J}})]+[2\times (100-{\rm{M}}{\rm{I}}{\rm{N}})]+(3\times {\rm{P}}{\rm{I}}{\rm{A}}{\rm{I}}{\rm{H}}),$$

where PROG was progressive motility, MAJ was major defects, MIN was minor defects and PIAIH was sperm with plasma and acrosome membrane integrity and high mitochondrial membrane potential.

#### Sperm quality assessment of selected-semen batches

The selected semen batches were evaluated by all the previously described analyses and also by flow cytometry using the BD Accuri C6^TM^ (Becton-Dickinson, San Jose, CA, USA) equipped with an argon laser (488 nm) and red laser (635 nm) both used to excite the samples. The following four filters were used in the analyses: 533/30 nm, 585 nm/40 nm, 675/25 nm and >670 nm. Particles and cellular debris were excluded from the acquisition using size and scatter properties and also by staining sperm samples with 2 µL of the nuclear dye Syto59 750 mM. Manual compensation analysis was performed adding controls: sperm samples were submitted to flash-freezing for plasma, acrosome and mitochondrial membrane controls^47^; for superoxide sperm generation controls, sperm samples were incubated with Antimycin A (20 µM) and DPI (diphenyleneiodonium chloride; 10 µM) at 37 °C for 1 hour^49^; for lipid peroxidation controls, sperm samples were incubated with ROS generator system (iron sulfate 4 mM, sodium ascorbate 20 mM and hydrogen peroxide 4 mM) at 37 °C for 30 minutes^48^; finally, for DNA fragmentation controls, sperm samples were exposed to hydrogen peroxide 10 mM for 1 hour^50^.

Acquisition rate was approximately 600 to 1,000 events/second, totalling 5,000 to 10,000 cells per analysis. Sperm concentration was adjusted to 5 × 10^6^ sperm/mL with TALP sperm for the incubation and adjusted to 2.5 × 10^6^ sperm/mL for flow cytometry analysis. For sperm plasma and acrosome membrane integrity, 2 µL of Syto59 750 mM, 1 µL of PI 0.5 mg/mL and 1 µL of FITC-PNA 37.5 µg/mL were added to 150 µL of diluted semen and incubated at 37 °C/10 minutes. For mitochondrial membrane potential evaluation, 2 µL of Syto59 750 mM, 1 µL of PI 0.5 mg/mL and 1 µL of JC-1 153 µM were added to 150 µL of diluted semen and incubated at 37 °C/10 minutes. For production of superoxide anion by mitochondria, 1.5 µL of MitoSOX 2 µM was added to 150 µL of diluted semen and incubated at 37 °C/30 minutes. After a 10-minute incubation, 1 µL of YoPro 7.5 µM was added; and at 20 minutes, 1.5 µL of Syto59 750 mM was added. For lipid peroxidation analysis, 1 µL of BODIPY 2 mM was added to 150 µL of diluted semen and incubated at 37 °C/30 minutes. At 20 minutes, 1 µL of PI 0.5 mg/mL and 2 µL of Syto59 750 mM were added. For DNA fragmentation, AO was used according to the protocol described by Simões *et al*.^51^.

#### Sperm miRNA levels

Two semen straws (0.25 mL) from the same batch were thawed (37 °C/30 seconds) and each 200 µL sample was placed into a Percoll gradient (45% and 90%) and centrifuged at 3,600 *g* for 7 minutes. The pellet was resuspended in PBS (10 mg/mL sodium chloride, 0.2 mg/mL potassium chloride, 1.44 mg/mL sodium phosphate dibasic, and 0.24 mg/mL potassium phosphate) and centrifuged twice at 520 *g* for 5 minutes. RNA extraction and purification were performed with miRNeasy Mini Kit^®^ (Qiagen, Hilden, Germany) according to the manufacturer’s instructions. RNA concentration and purity were determined by NanoDrop™ 1000 (Thermo Scientific, Massachusetts, USA) spectrophotometer. Complementary DNA (cDNA) was generated using miScript RT Kit^®^ (Qiagen, Hilden, Germany) by adding a volume of approximately 100 ng of total RNA to HiFlex buffer, 10 × nucleic acid mix, reverse transcriptase enzyme and RNAse/DNAse-free water followed by incubation at 37 °C/1 hour and 95 °C/5 minutes. Next, the abundance of a customized panel of 380 bovine-specific miRNAs (Supplementary Table S11) was evaluated by qPCR. The primer sequences were obtained from the miRBase database (www.mirbase.org). Bta-miR-99b and -425-5p, consistently detected among the groups according to the NormFinder platform^18,52,53^, were used to generate the relative levels. The PCR mix was prepared using SYBR Green^®^ (Qiagen, Hilden, Germany). A 384-well plate was used for PCR and the reaction was performed on QuantStudio^TM^ 6 Flex (Thermo Fisher Scientific) with the following cycle conditions: an initial incubation of 95 °C/15 minutes and 45 cycles of 94 °C/15 seconds, 55 °C/30 seconds and 70 °C/30 seconds. The melting-curve was used to confirm the amplification of a single product.

#### *In vitro* embryo production

HF (n = 3) and LF (n = 3) selected batches were used to produce embryos. *In vitro* embryo production was performed in triplicate for each bull to give a total of nine HF and LF replicates. Parthenogenetic embryos and mature oocytes were produced in all replicates. Incubator conditions for oocytes, sperm cells and embryos were maintained at 38.5 °C during all steps under 5% CO_2_, air (20% oxygen) with saturating humidity. For embryo production, bovine ovaries were collected from a local slaughterhouse and transported at 35 °C in thermal bottles filled with sterile saline solution 0.9%. In the laboratory, ovarian follicles (3 to 6 mm) were aspirated with 18 G needles. The evaluation of cumulus–oocyte complexes (COCs) was performed under a stereomicroscope (SMZ 745T model, Nikon, Tokio, Japan). Then, COCs were washed in tissue culture medium 199 (TCM 199, Gibco, Thermo Scientific) supplemented with 10% extracellular vesicle-depleted foetal bovine serum (EV-free FBS; extracellular vesicle-depletion was performed by ultracentrifugation at 120,000 *g* for 16 hours), 22 µg/mL sodium pyruvate and 50 µg/mL gentamicin. Only grade I and grade II COCs^54^ were selected and washed in maturation medium [TCM 199 supplemented with 26 mM sodium bicarbonate, 10% EV-free FBS, 22 µg/mL sodium pyruvate, 50 µg/mL gentamicin, 0.5 µg/mL follicle-stimulating hormone (FSH; Folltropin^TM^, Bioniche Animal Health, Belleville, Canada) and 5 U/mL human chorionic gonadotropin (hCG; Vetecor^TM^, Hertape Calier, London, England)]. A total of 20 to 25 COCs were placed in 90 µL of maturation medium covered with mineral oil for 22 hours. After a period of *in vitro* maturation (IVM), oocytes were divided into three groups: parthenogenetic embryos (control, without sperm cells) and *in vitro* fertilized embryos with sperm from high and low fertility bulls; also, pools of five mature oocytes were collected from each replicate. For parthenogenetic embryos, presumptive mature oocytes were completely denuded with 0.2% hyaluronidase in PBS and were evaluated for extrusion of the first polar body. Only mature oocytes (oocytes that present extrusion of first polar body) were submitted to parthenogenetic activation (at 26 hours post IVM) by incubation in 5 µM ionomycin in HEPES-buffered TCM-199 for 5 minutes and 2 mM 6-dimethylaminopurine (6-DMAP) diluted in modified synthetic oviduct fluid (SOF) for 3 hours^55^. For *in vitro* fertilization (IVF), presumptive mature oocytes were incubated for 18 hours in IVF medium composed of Tyrode’s lactate stock supplemented with 50 µg/mL gentamicin, 22 µg/mL sodium pyruvate, 40 µg/mL PHE (2 mM D-penicillamine, 1 mM hypotaurine and 245 µM epinephrine), 5.5 IU/mL heparin and 6 mg/mL bovine serum albumin (BSA) and sperm cells from frozen–thawed semen from each bull with either high or low fertility. Each batch was processed on a Percoll gradient (45% and 90%) to obtain sperm cells at a concentration of 1 × 10^6^ sperm/mL. Presumptive zygotes from each group were divided into drops for evaluation and collection of zygotes, two-cell embryos and blastocysts. The presumptive zygotes were completely denuded using a pipette and were cultured in synthetic oviductal fluid with amino acids, sodium citrate and inositol (SOF) supplemented with 5 mg/mL BSA, 22 µg/mL sodium pyruvate, 50 µg/mL gentamicin and 2.5% EV-free FBS. Zygotes were evaluated and collected 14 to 16 hours post activation (hpa) or 16 to 18 hours post insemination (hpi; second polar body extrusion evaluation); two-cell embryos were evaluated and collected 20 to 22 hpa or 28 to 30 hpi. Cleavage rates were assessed on day-4 of development and blastocysts were evaluated and collected on day-7. Samples were either stored at −80 °C for miRNA and mRNA analyses, or fixed in paraformaldehyde 4% in PBS with 0.1% PVP (PBS + PVP) for imaging analysis.

#### Embryo mRNA and miRNA levels

Prior to being snap frozen, the zona pellucida of zygotes and two-cell embryos was removed by enzymatic digestion with pronase 0.5% (Protease from *Streptomyces griseus*). Nine pools of five embryos and three pools of five oocytes were snap frozen in PBS + PVP and used to evaluate miRNA and mRNA levels. RNA extraction and purification were performed using miRNeasy Mini Kit^®^ (Qiagen, Hilden, Germany) according to the manufacturer’s instructions; concentration and purity were determined by NanoDrop™ 1000 (Thermo Scientific, Massachusetts, USA) spectrophotometer. For miRNA analyses, cDNA was generated using the miScript RT Kit^®^ (Qiagen, Hilden, Germany) as previously described. Only miRNAs differentially abundant between sperm cells from HF and LF bulls were evaluated by qPCR. Bta-miR-99b and RNU43 snoRNA were consistently detected among the groups and were used to evaluate the relative levels of these miRNAs. The qPCR reaction was performed under the same conditions detailed before. For mRNA analyses, cDNA was generated using High-Capacity Kit^®^ (Applied Biosystem, Foster City, California, USA) according to the manufacturer’s instructions. Next, the levels of miRNA target genes that were different in sperm cells and zygotes were analysed by qPCR. Target genes were determined by first evaluating the homology between bovine miRNAs and human miRNAs using the miRBase platform (http://www.mirbase.org/). Strongly validated target genes of human miRNAs were then researched on the miRTarBase platform (http://mirtarbase.mbc.nctu.edu.tw/php/index.php). Conservation of the 3′ untranslated region (UTR) of the target gene was also investigated using the TargetScanHuman platform (http://www.targetscan.org/vert_72/). Target gene primer sequences (Supplementary Table S12) were designed based on GenBank and sequence specificity was verified by Nucleotide Blast of the NCBI platform (https://blast.ncbi.nlm.nih.gov/). β*-*Actin was used as a housekeeping gene. qPCR was performed with 8.5 ng of RNA per reaction following the manufacturer’s instructions for the Power Sybr^®^ kit (Applied Biosystem, Foster City, California, USA). The PCR reaction was performed on a QuantStudio^TM^ 6 Flex (Thermo Fisher Scientific) with the following cycle conditions: an initial incubation of 95 °C/10 minutes and 40 cycles of 95 °C/15 seconds and 60 °C/60 seconds. The melting-curve was used to confirm the amplification of a single product.

#### Blastocyst qualitative analysis

On day-7 following IVF or activation, blastocysts were evaluated according to stage (early blastocyst, blastocyst, expanded and hatched). Blastocysts were also collected and fixed for immunofluorescence proliferation evaluation: eight blastocysts from the HF group and 10 blastocysts from the LF group were fixed for 12 minutes in 4% PFA in PBS + PVP. Next, blastocysts were permeabilized with 1% Triton X-100 for 20 minutes and blocked with 5% BSA and 22 mg/mL glycine for 1 hour at room temperature. They were then incubated overnight at 4 °C with a rabbit anti-Ki-67 (1:100, 5500134, Sigma-Aldrich) primary antibody. Primary antibody was detected with anti-rabbit IgG AlexaFluor 488 (1:500, A11008, Thermo Fisher Scientific). Finally, the embryos were stained with 10 µL/mL Hoechst/15 minutes. Blastocyst slides were mounted with Prolong Antifade^®^ (Thermo Fisher Scientific). The images were captured by confocal microscopy SP5 (Leica, Wetzlar, Germany) using argon (488 nm) and diode (405 nm) laser under 400× magnification. A negative control was performed for each set of analyses. Blastocyst diameter, number of cells stained with Hoechst 33342 and stained with anti-Ki-67 were analysed using Z-project on ImageJ^®^ (NIH, https://imagej.nih.gov/ij/). The percentage of proliferation was determined by calculating the number of cells stained with Ki-67 divided by the total of cells stained with Hoechst. The cell number was determined by counting Hoechst-stained cells. The cell division number (n) was calculated using the formula: Log_2_(n) = cell number.

#### Embryo kinetic evaluation

Early embryonic kinetics of HF (n = 3) and LF (n = 3) selected batches were evaluated by subjecting semen samples for *in vitro* embryo production as previously described with an IVF duration of 8 hours. Embryos were evaluated with regard to the two-cell and four-cell rates 28 to 30 hpi and 40 to 42 hpi, respectively.

### Experiment 2: Higher level of miR-216b is associated with polyspermic zygotes

#### Semen samples and experimental design

For this experiment, in order to select semen samples that present the greatest difference in miR-216b relative levels, frozen–thawed semen from two bulls (one semen sample from one HF bull and one semen sample from one LF bull) were selected based on the results of experiment 1. For validation of polyspermy induction, the HF and LF semen samples selected were pooled and tested to give a total of seven replicates. Afterwards, a total of five HF and LF semen replicates were individually evaluated to determine the relative levels of miR-216b in polyspermic embryos.

#### Validation of polyspermy induction and polyspermy evaluation

To verify polyspermy induction rates, a sperm concentration of 1 × 10^6^ sperm/mL per IVF drop was considered the control and a concentration of 8 × 10^6^ sperm/mL was considered polyspermy induced. Both concentrations were tested in seven replicates using pools of the selected semen batches from HF and LF bulls. IVM and IVF were performed as described before, with the exception that the sperm concentration in the IVF drop varied according to the group. Second polar body-positive embryos were collected and fixed 12 hpi. Embryo fixation was performed with 4% PFA in PBS + PVP and embryos were stained with 10 µL/mL Hoechst/15 minutes. Slides with embryos were mounted with Prolong Antifade^®^ and the number of pronuclei was evaluated by epifluorescence microscopy (Nikon, model 80i, Tokyo, Japan) at 600× magnification using UV-2E/C filter (excitation 340–380 nm emission 435–485 nm).

#### MiR-216b relative level in polyspermic embryos

HF and LF semen samples selected according to miR-216b relative level were used to perform five replicates of IVF with adjusted sperm concentrations for control (1 × 10^6^ sperm/mL) and polyspermy induction (8 × 10^6^ sperm/mL). Embryos were collected 16 to 18 hpi and stored at −80 °C to evaluate the abundance of miR-216b. Five pools of 15 embryos were subjected to RNA extraction and the relative levels of miR-216b were verified by qPCR following the protocols previously described.

### Statistical analysis

Statistical Analysis System (SAS Institute Inc, 9.3) software was used for all statistical analysis. Shapiro-Wilk was performed to evaluate the normality of data. When necessary, data were transformed or outliers were removed. The significance level was P < 0.05 except for miRNAs in sperm cells where P ≤ 0.10. Analysis of variance (ANOVA) using the mixed procedure was performed to evaluate sperm characteristics, the miRNA and mRNA relative levels and blastocyst quality features. The Tukey test was used when the parthenogenetic and/or mature oocyte groups were added. The Chi-Square frequency test was performed to evaluate embryo development and polyspermy rates. Analysis of variance (ANOVA) using the mixed procedure respecting the factorial design was performed to evaluate the relative level of miR-216b in polyspermic embryos (Experiment 2). For molecular analysis, ΔCT was considered for statistical analysis and relative level data were presented as 2^−ΔCT^ as shown in the graphics, tables and Venn Diagram (http://bioinfogp.cnb.csic.es/tools/venny/). Exclusively miRNA detection was based on the miRNA detection in at least two semen samples. Non-detection was considered when it appeared in only one or none of the samples.

## Supplementary information

Supplementary Information

## Data Availability

The datasets generated and/or analysed during the current study are available from the corresponding author on reasonable request.
