# Supplementary material for: Sperm-borne miR-216b modulates cell proliferation during early embryo development via K-RAS
Source: Sci Rep. 2019 Jul 17;9:10358. doi: 10.1038/s41598-019-46775-8 (PMC6637201; doi:10.1038/s41598-019-46775-8)
Supplement: Supplementary file 1 — Supplementary Information [file 41598_2019_46775_MOESM1_ESM.pdf]

Title: Sperm-borne miR-216b modulates cell proliferation during early embryo development via *K-RAS*

Authors: Maíra Bianchi Rodrigues Alves[1], Rubens Paes de Arruda[1], Tiago Henrique Camara De Bem[2], Shirley Andrea Florez-Rodriguez[1], Manoel Francisco de Sá Filho[1,3], Clémence Belleannée[4], Flávio Vieira Meirelles[2], Juliano Coelho da Silveira[2], Felipe Perecin[2], and Eneiva Carla Carvalho Celeghini[1]*

[1]Department of Animal Reproduction, School of Veterinary Medicine and Animal Science, Universidade de São Paulo, Pirassununga, São Paulo, Brazil.

[2]Department of Veterinary Medicine, School of Animal Science and Food Engineering, Universidade de São Paulo, Pirassununga, São Paulo, Brazil.

[3]Alta Genetics of Brazil, Uberaba, Minas Gerais, Brazil.

[4]Department of Obstetrics, Gynecology and Reproduction, Université Laval, Quebec, Quebec, Canada.

*Correspondence: Professor Eneiva Carla Carvalho Celeghini. E-mail: celeghin@usp.br. Street Duque de Caxias Norte, 225 – Pirassununga – Brazil – Zip code 13635-900. Tel: +55 19 3565 4242.

Supplementary Table S1. Mean and SEM of sperm quality characteristics evaluated in three commercial semen batches from each bull. [a,b] Different letters on the same line indicate statistical difference ($P < 0.05$) between fertility groups. [1]High fertility; [2] Low fertility; [3]Sperm with plasma and acrosome membrane integrity and high mitochondrial membrane potential; [4]Production of ROS (reactive oxygen species). SEM: standard error of the mean.

Sperm quality analyses of all straws	Fertility groups	
	HF[1] (n=9)	LF[2] (n=9)
Sperm conventional analyses		
Straw volume (mL)	0.25	0.25
Subjective motility (%)	66.4±1.1	63.3±1.1
Vigor (1-5)	3.4±0.1	3.3±0.1
Sperm concentration (×10^6 sperm/mL)	100.9±5.4	90.8±4.7
Major defects (%)	18.7±2.0[b]	25.0±0.9[a]
Minor defects (%)	4.6±1.1	4.2±0.7
Total defects (%)	23.4±2.6	29.2±1.1
Total of sperm/straw (×10^6)	25.2±1.4	22.7±1.2
Total of motility sperm/straw (×10^6)	17.1±0.8[a]	13.5±0.8[b]
Sperm motility characteristics		
Total motility (%)	76.7±1.9	71.9±1.7
Progressive motility (%)	54.7±2.0	52.5±1.7
Rapid cells (%)	70.5±1.7	66.7±1.6
Curvilinear velocity (VCL; µm/s)	145.8±2.6[b]	172.8±3.7[a]
Progressive velocity (VSL; µm/s)	78.3±2.7	83.9±1.9
Path velocity (VAP; µm/s)	97.4±1.5[b]	105.4±1.6[a]
Linearity (LIN; %)	53.7±1.6[a]	48.7±1.0[b]
Straightness (STR; %)	81.1±1.4	80.6±0.9
Wobble (WOB; %)	66.0±1.0[a]	60.4±0.8[b]
Lateral head displacement (ALH; µm)	2.7±0.04[b]	3.4±0.1[a]
Beat cross frequency (BCF; Hz)	22.3±0.3	23.1±0.5
Sperm membranes integrity, mitochondrial membrane function and ROS production		
PIAIH[3] (%)	42.7±2.4	42.1±1.8
Plasma membrane integrity (%)	44.4±2.4	46.8±2.6
Acrosome membrane integrity (%)	53.4±2.4	57.7±1.4
High mitochondrial membrane potential (%)	58.0±3.2	52.6±2.8
ROS[4] (%)	6.1±1.3[b]	10.6±1.5[a]

Supplementary Table S2. Individual characteristics and values obtained for all three commercial semen batches from each bull in order to select the best batch from each bull. Bold text indicates the selected batch of each bull. HF: high fertility; LF: low fertility. [1]Progressive motility; [2]Major defects; [3]Minor defects; [4]Sperm with plasma and acrosome membranes integrity and high mitochondrial membrane potential; [5]Value obtained by the formula: (1 × PROG) + [(3 × (100 − MAJ)] + [(2 × (100 − MIN)] + (3 × PIAIH).

Bull	Batch	PROG[1] (%)	MAJ[2] (%)	MIN[3] (%)	PIAIH[4] (%)	Value[5]
HF-1	**1**	**54.1**	**20.7**	**9.2**	**58.2**	**648.2**
	2	52.8	13.2	2.4	34.2	611.0
	3	43.2	22.0	7.2	37.2	574.4
HF-2	1	55.0	7.7	1.5	45.7	666.0
	2	59.3	16.2	1.7	49.0	654.3
	**3**	**68.5**	**9.0**	**3.0**	**43.5**	**666.0**
HF-3	**1**	**49.2**	**20.0**	**7.2**	**45.2**	**610.4**
	2	60.0	33.0	4.7	41.7	576.7
	3	50.0	26.7	4.7	29.7	549.6
LF-1	**1**	**49.7**	**23.2**	**3.7**	**37.7**	**585.8**
	2	48.4	19.7	4.0	33.7	582.4
	3	46.0	23.2	3.0	35.7	577.5
LF-2	1	49.4	27.7	3.5	39.2	576.9
	2	57.5	26.7	6.0	38.5	580.9
	**3**	**63.1**	**27.2**	**6.0**	**40.5**	**591.0**
LF-3	1	51.1	30.7	4.0	46.7	591.1
	2	44.5	24.2	4.2	49.2	611.1
	**3**	**62.5**	**22.**	**3.2**	**57.2**	**661.7**

Supplementary Table S3. Mean and SEM of sperm quality characteristics evaluated in each of the three commercial semen batches from HF-1, HF-2 and HF-3 bulls. Bold text indicates the selected batch. [1]High fertility; [2]Sperm with plasma and acrosome membranes integrity and high mitochondrial membrane potential; [3]Production of ROS (reactive oxygen species). SEM: standard error of the mean.

Sperm quality	Semen batches from high fertility bulls								
	**HF[1]-1-1**	HF[1]-1-2	HF[1]-1-3	HF[1]-2-1	HF[1]-2-2	**HF[1]-2-3**	**HF[1]-3-1**	HF[1]-3-2	HF[1]-3-3
Total sperm (×10^6)	19.4±5.0	33.7±1.9	26.2±3.1	29.9±4.1	21.6±2.2	28.1±5.0	20.0±1.2	27.5±3.1	24.4±5.0
Total sperm with motility (×10^6)	12.9±2.9	21.0±0.3	16.3±1.3	18.2±4.2	13.9±0.3	19.1±4.1	14.0±0.9	19.2±2.2	15.6±2.03
Subjective motility (%)	67.5±2.5	62.5±2.5	62.5±2.5	67.5±7.5	65.0±5.0	67.5±2.5	70.0±0.0	70.0±0.0	65.0±5.0
Vigor (1-5)	3.2±0.2^{ab}	3.5±0.0^{a}	2.5±0.0^{b}	3.5±0.0	3.5±0.0	3.7±0.2	3.5±0.0	3.5±0.0	3.5±0.0
Sperm concentration (×10^6 sperm/mL)	77.5±20.0	135.0±7.5	105.0±12.5	103.7±16.2	86.2±8.7	112.5±20.0	80.0±5.0	110.0±12.5	97.5±2
Major defects (%)	20.7±3.2	13.2±2.2	22.0±4.0	7.7±0.2^{b}	16.2±1.2^{a}	8.7±2.2^{ab}	20.0±5.0	33.0±1.5	26.7±2.2
Minor defects (%)	9.2±6.7	2.4±1.4	7.2±5.7	1.5±0.0	1.7±0.7	3.0±2.0	7.2±5.2	4.7±0.7	4.7±3.2
Total defects (%)	30.0±3.5	15.5±3.5	29.2±9.7	9.2±0.2	18.0±2.0	11.7±4.2	25.2±10.2	37.7±2.2	31.5±5.5
Total motility (%)	81.2±4.6	75.1±6.0	72.2±4.0	70.5±1.6	76.5±2.9	80.5±2.2	84.9±1.6	85.4±0.8	63.5±7.5
Progressive motility (%)	54.1±0.8	52.8±1.3	43.2±12.5	55.0±0.9^{b}	59.3±3.1^{ab}	68.2±1.5^{a}	49.2±2.1	60.0±4.5	50.0±1.3
Rapid cells (%)	76.0±4.2	70.1±4.5	65.2±1.0	64.1±0.9^{b}	69.9±2.4^{ab}	75.5±0.4^{a}	75.1±1.6	79.3±2.5	59.0±7.7
Curvilinear velocity (VCL; µm/s)	160.3±2.8	158.1±7.1	136.5±11.1	149.7±12.7	146.9±1.3	139.8±0.7	133.9±2.7^{b}	140.0±1.1^{ab}	146.7±0.4^{a}

Progressive velocity (VSL; µm/s)	75.9±1.9	80.0±80.1	62.6±12.9	88.5±4.9	81.9±0.5	87.2±4.6	63.8±1.1	80.5±5.7	84.5±7.8
Path velocity (VAP; µm/s)	99.1±1.0	100.5±5.2	81.7±7.7	102.7±5.9	97.0±0.2	99.4±2.9	86.4±0.05	99.5±4.5	98.7±4.9
Linearity (LIN; %)	47.3±0.3	50.4±2.8	45.3±5.7	59.3±1.7	55.8±0.8	62.3±2.9	47.7±1.8	57.5±3.6	57.6±5.2
Straightness (STR; %)	76.5±1.1	79.4±3.9	75.7±8.6	86.2±0.2	84.4±0.3	87.6±2.0	73.8±1.3	80.8±2.1	85.4±3.6
Wobble (WOB; %)	61.8±0.4[ab]	63.5±0.4[a]	59.8±0.7[b]	68.7±1.8	66.0±0.7	71.1±1.7	64.7±1.1	71.0±2.6	67.2±3.1
Lateral head displacement (ALH; µm)	2.8±0.05	2.7±0.05	2.7±0.1	2.7±0.2	2.7±0.0	2.4±0.05	2.6±0.1	2.4±0.0	2.7±0.1
Beat cross frequency (BCF; Hz)	21.8±0.8	23.6±1.4	21.0±1.4	23.6±0.4	23.2±0.5	23.0±0.05	20.5±0.05[b]	20.9±0.2[b]	22.7±0.1[a]
PIAIH[2] (%)	58.2±2.7[a]	34.2±3.2[b]	37.2±1.7[b]	45.7±8.7	49.0±8.0	43.2±4.7	45.2±3.7[a]	41.7±1.7[a]	29.7±1.7[b]
Plasma membrane integrity (%)	61.5±0.0[a]	35.2±3.2[b]	39.0±2.5[b]	47.0±1	49.7±8.7	44.7±4.7	48.2±3.7[a]	43.7±1.7[a]	30.5±1.0[b]
Acrosome membrane integrity (%)	67.2±6.7[a]	41.2±0.7[b]	44.7±0.7[b]	55.2±4.7	60.2±9.7	55.0±1.0	60.0±6.0	56.0±2.0	41.0±2.0
High mitochondrial membrane potential (%)	78.0±6.5	54.7±11.2	73.0±3.5	53.2±11.2	56.7±8.2	52.0±9.5	57.7±5.2	57.5±6.0	39.0±3.5
ROS[3] (%)	5.5±0.5	16.5±5.5	5.5±1.5	5.5±3.5	4.5±3.5	3.5±2.5	6.50±5.5	39.5±39.5	4.5±1.5

Supplementary Table S4. Mean and SEM of sperm quality characteristics evaluated in each of the three commercial semen batches from LF-1, LF-2 and LF-3 bulls. Bold text indicates the selected batch. [1]Low fertility; [2]Sperm with plasma and acrosome membranes integrity and high mitochondrial membrane potential; [3]Production of ROS (reactive oxygen species). SEM: standard error of the mean.

Sperm quality	Semen batches from low fertility bulls								
	**LF[1]-1-1**	LF[1]-1-2	LF[1]-1-3	LF[1]-2-1	LF[1]-2-2	**LF[1]-2-3**	LF[1]-3-1	LF[1]-3-2	**LF[1]-3-3**
Total sperm ($\times 10^6$)	21.9±1.9	27.5±5.6	23.7±3.1	15.3±0.3	20.3±2.2	17.2±1.6	26.2±1.2	25.0±1.2	27.2±1.6
Total sperm with motility ($\times 10^6$)	11.9±0.1	16.8±4.7	13.9±3.6	9.2±0.2	11.5±0.3	10.3±0.93	17.1±0.8	16.8±0.2	18.3±0.4
Subjective motility (%)	55.0±5.0	60.0±5.0	57.5±7.5	60.0±0.0	57.5±7.5	60.0±0.0	65.0±0.0	67.5±2.5	67.5±2.5
Vigor (1-5)	3.5±0.0	3.2±0.2	3.2±0.2	3.2±0.2	2.7±0.2	3.0±0.0	3.5±0.0	3.5±0.0	3.5±0.0
Sperm concentration (x10^6 sperm/mL)	87.5±7.5	110.0±22.5	95.0±12.5	61.25±1.2	81.2±8.7	68.7±6.2	105.0±5.0	100.0±5.0	108.7±6.2
Major defects (%)	23.2±1.2	19.7±0.7	23.2±1.2	27.7±1.2	26.7±2.7	27.2±4.2	30.7±0.2[a]	24.2±0.2[b]	22.4±1.5[b]
Minor defects (%)	3.7±1.2	4.0±1.5	3.00±1.5	3.5±2.5	6.0±4.5	6.0±4.0	4.0±2.0	4.2±2.7	3.2±1.30.0
Total defects (%)	27.0±0.0	23.7±2.2	26.25±2.7	31.2±1.2	32.7±7.2	33.2±0.2	34.7±1.7	28.5±2.5	25.6±2.8
Total motility (%)	63.8±2.4	67.4±3.9	63.35±2.1	74.5±0.9	69.6±1.7	76.4±3.7	76.8±6.2	73.8±6.2	81.6±2.9
Progressive motility (%)	49.7±3.9	48.4±5.5	46.05±1.8	49.4±3.5	57.5±0.4	63.1±2.7	51.1±0.3[b]	44.5±0.5[c]	62.5±1.6[a]
Rapid cells (%)	60.7±3.3	61.5±7.3	59.50±2.1	66.0±2.1	67.5±2.2	70.3±4.0	70.3±4.1	67.4±3.2	76.7±2.5
Curvilinear velocity (VCL; µm/s)	185.5±2.7	184.9±5.1	186.9±7.6	150.4±7.4	160.0±7.3	150.7±3.7	181.1±6.5	175.6±0.4	179.8±6.6

Progressive velocity (VSL; µm/s)	86.9±0.8	84.6±3.0	87.7±6.1	70.5±7.0	87.9±0.6	81.6±3.9	86.9±7.9	75.4±1.5	93.5±1.8
Path velocity (VAP; µm/s)	105.3±0.4	104.8±3.0	108.5±6.4	90.8±9.0	105.2±2.0	94.7±3.1	110.4±4.4	102.8±1.1	114.2±3.1
Linearity (LIN; %)	46.8±0.2	45.7±0.3	46.9±1.4	46.7±2.3	55.1±2.9	54.1±1.2	47.90±2.0	43.0±0.8	52.0±0.8
Straightness (STR; %)	82.6±0.4	80.7±0.6	80.8±0.85	77.6±0.05	83.6±2.1	86.1±1.2	78.60±4.0	73.4±0.7	81.9±0.6
Wobble (WOB; %)	56.8±0.6	57.7±0.1	58.0±1.1	60.2±3.0	65.8±1.8	62.8±0.5	60.90±0.2[ab]	58.6±0.5[b]	63.5±0.6[a]
Lateral head displacement (ALH; µm)	3.8±0.1	4.0±0.2	3.9±0.2	2.8±0.05	2.7±0.05	2.8±0.0	3.80±0.2	3.5±0.2	3.3±0.1
Beat cross frequency (BCF; Hz)	23.8±0.5	21.7±1.3	23.0±1.2	22.0±1.1	26.6±1.1	26.3±0.8	21.10±1.1	20.9±0.8	22.4±0.2
PIAIH[2] (%)	37.7±0.2	33.7±1.7	35.7±2.7	39.2±2.7	38.5±0.0	40.5±0.5	46.75±5.7	49.2±0.2	57.2±3.7
Plasma membrane integrity (%)	43.0±3.5	34.7±2.7	38.7±2.2	41.2±4.2	40.2±1.2	41.2±0.7	66.25±5.7	54.2±2.2	61.2±0.7
Acrosome membrane integrity (%)	60.5±1.5	57.7±1.2	57.7±1.7	56.0±2.5	53.5±4.5	51.7±0.2	55.25±8.2	60.5±3.5	66.0±7.0
High mitochondrial membrane potential (%)	41.2±1.2	35.7±1.7	42.0±2.5	49.5±3.0	49.5±4.5	57.2±3.2	68.50±2.5	64.0±3.0	65.7±1.2
ROS[3] (%)	7.0±0.0	7.5±4.5	8.7±6.2	11.5±0.5	6.0±5.0	4.5±1.5	15.5±4.5	14.5±1.5	20.5±3.5

Supplementary Table S5. Mean and SEM of relative levels of the panel of 380 miRNAs detected in bovine gametes: sperm cells from high (HF) and low fertility (LF) bulls and in mature oocytes. SEM: standard error of the mean.

miRNAs	Sperm cells						Mature oocytes (n=3)		
	HF (n=3)			LF (n=3)					
Detected in both groups (HF and LF) of sperm cells									
bta-let-7a-3p	0.0082	±	0.0029	0.0052	±	0.0064	.	±	.
bta-let-7a-5p	0.0787	±	0.0399	0.0820	±	0.0179	0.0060	±	0.0017
bta-let-7c	0.0655	±	0.0376	0.0462	±	0.0203	0.0056	±	0.0034
bta-let-7d	0.0275	±	0.0122	0.0283	±	0.0087	0.0067	±	0.0018
bta-let-7e	0.1503	±	0.0720	0.1566	±	0.0185	0.0198	±	0.0155
bta-let-7f	0.0064	±	0.0042	0.0104	±	0.0054	0.0021	±	0.0015
bta-let-7g	0.0245	±	0.0080	0.0114	±	0.0138	0.0042	±	0.0022
bta-let-7i	0.0116	±	0.0091	0.0065	±	0.0037	0.0072	±	0.0045
bta-miR-7	0.0403	±	0.0391	0.0234	±	0.0192	0.0049	±	0.0012
bta-miR-9-5p	0.0225	±	0.0145	0.0211	±	0.0146	0.0100	±	0.0074
bta-miR-10a	0.3017	±	0.1970	0.4891	±	0.0931	0.0454	±	0.0070
bta-miR-10b	0.0049	±	0.0028	0.0059	±	0.0032	0.0429	±	0.0074
bta-miR-15a	0.7730	±	0.7098	1.0576	±	0.9029	0.0422	±	0.0035
bta-miR-15b	1.5945	±	1.5854	1.7931	±	1.6892	0.0134	±	0.0051
bta-miR-16a	2.2002	±	1.8295	2.5497	±	2.7160	0.1019	±	0.0280
bta-miR-16b	5.3977	±	4.7208	4.0805	±	2.1031	0.2727	±	0.2049
bta-miR-17-5p	0.0725	±	0.0696	0.0549	±	0.0341	0.0171	±	0.0024
bta-miR-18a	0.0863	±	0.0674	0.0838	±	0.0811	0.0102	±	0.0044
bta-miR-18b	0.0289	±	0.0261	0.0212	±	0.0202	0.0023	±	0.0016
bta-miR-19a	0.0286	±	0.0337	0.0134	±	0.0198	0.0377	±	0.0034
bta-miR-19b	0.0349	±	0.0414	0.0158	±	0.0150	0.0462	±	0.0296
bta-miR-20a	0.7040	±	0.6306	0.4886	±	0.3146	0.0443	±	0.0072
bta-miR-20b	0.2230	±	0.2159	0.2580	±	0.1985	0.0225	±	0.0039
bta-miR-21-5p	0.0134	±	0.0128	0.0133	±	0.0020	0.0037	±	0.0028

bta-miR-22-5p	0.0018	±	0.0014	0.0026	±	0.0037	0.0120	±	0.0038
bta-miR-23a	0.0625	±	0.0436	0.0895	±	0.0414	0.0119	±	0.0055
bta-miR-23b-3p	0.0017	±	0.0014	0.0031	±	0.0013	0.0526	±	0.0702
bta-miR-24-3p	0.0235	±	0.0144	0.0190	±	0.0238	0.0142	±	0.0032
bta-miR-25	0.1769	±	0.0393	0.2059	±	0.1237	0.0092	±	0.0045
bta-miR-26a	0.0563	±	0.0559	0.0460	±	0.0361	0.0848	±	0.0746
bta-miR-26b	0.0032	±	0.0003	0.0032	±	0.0031	0.0060	±	0.0071
bta-miR-27a-3p	0.2037	±	0.2382	0.1972	±	0.2456	0.0522	±	0.0674
bta-miR-27b	0.0973	±	0.0979	0.0931	±	0.0757	0.0795	±	0.0772
bta-miR-28	0.0047	±	0.0014	0.0039	±	0.0017	.	±	.
bta-miR-29a	0.1277	±	0.1156	0.1502	±	0.1266	.	±	.
bta-miR-29b	0.0215	±	0.0137	0.0236	±	0.0292	0.0027	±	0.0023
bta-miR-29c	0.1176	±	0.1196	0.1621	±	0.1269	0.0027	±	0.0008
bta-miR-29d-3p	0.0157	±	0.0066	0.0176	±	0.0181	.	±	.
bta-miR-29d-5p	0.0012	±	0.0014	0.0027	±	0.0025	0.0027	±	0.0012
bta-miR-29e	0.0059	±	0.0033	0.0094	±	0.0063	.	±	.
bta-miR-30a-5p	0.0515	±	0.0559	0.0891	±	0.0726	0.0143	±	0.0075
bta-miR-30b-5p	0.0627	±	0.0710	0.0600	±	0.0516	0.0468	±	0.0234
bta-miR-30c	0.0508	±	0.0584	0.0414	±	0.0491	0.1560	±	0.2161
bta-miR-30d	0.0606	±	0.0541	0.0517	±	0.0568	0.0136	±	0.0089
bta-miR-30e-5p	0.0652	±	0.0615	0.0634	±	0.0689	0.0164	±	0.0013
bta-miR-30f	0.0131	±	0.0024	0.0037	±	0.0030	0.0151	±	0.0088
bta-miR-33b	0.0004	±	0.0002	0.0002	±	0.0000	0.0007	±	0.0006
bta-miR-34a	0.0342	±	0.0412	0.0146	±	0.0034	0.0070	±	0.0070
bta-miR-34b	0.3081	±	0.3086	0.5956	±	0.6895	0.0157	±	0.0056
bta-miR-34c	0.3413	±	0.3560	0.6209	±	0.7448	0.0203	±	0.0195
bta-miR-92a	0.0264	±	0.0112	0.0332	±	0.0128	0.0624	±	0.0259
bta-miR-92b	0.0906	±	0.0335	0.0621	±	0.0093	9.8715	±	2.7442
bta-miR-93	0.6598	±	0.5074	0.8192	±	0.6432	0.0103	±	0.0007
bta-miR-95	0.0026	±	0.0011	0.0041	±	0.0043	.	±	.
bta-miR-99a-5p	1.0856	±	0.7910	1.7422	±	1.0775	0.0043	±	0.0037

bta-miR-99b	0.9146	±	0.3110	0.7662	±	0.2567	4.4615	±	0.8911
bta-miR-99b	0.9223	±	0.2811	0.8128	±	0.2058	5.7071	±	0.4555
bta-miR-100	0.9619	±	0.6599	1.2005	±	0.9492	0.0136	±	0.0020
bta-miR-101	0.0882	±	0.1035	0.1185	±	0.1503	0.0057	±	0.0034
bta-miR-103	0.0598	±	0.0553	0.0660	±	0.0652	0.0065	±	0.0047
bta-miR-105a	0.0014	±	0.0015	0.0014	±	0.0010	0.0075	±	0.0089
bta-miR-105b	0.0034	±	0.0011	0.0015	±	0.0011	0.0137	±	0.0083
bta-miR-106a	0.6168	±	0.6309	0.5302	±	0.4100	0.0466	±	0.0238
bta-miR-106b	0.1893	±	0.1984	0.1591	±	0.1428	0.0124	±	0.0061
bta-miR-122	0.0064	±	0.0040	0.0140	±	0.0030	0.0044	±	0.0033
bta-miR-122	0.0079	±	0.0052	0.0121	±	0.0042	0.0036	±	0.0031
bta-miR-124a	0.0002	±	0.0001	0.0009	±	0.0008	0.0065	±	0.0030
bta-miR-124b	0.0009	±	0.0006	0.0003	±	0.0001	0.0073	±	0.0037
bta-miR-125a	0.0045	±	0.0027	0.0079	±	0.0099	0.0117	±	0.0169
bta-miR-125b	0.5888	±	0.5419	0.7015	±	0.8170	0.0044	±	0.0007
bta-miR-1260b	39.7555	±	33.8572	35.3772	±	7.6421	22.6233	±	17.8693
bta-miR-126-3p	0.0090	±	0.0019	0.0062	±	0.0026	0.0919	±	0.1301
bta-miR-126-5p	0.0103	±	0.0030	0.0063	±	0.0008	0.0869	±	0.1112
bta-miR-127	0.0080	±	0.0044	0.0045	±	0.0010	0.0416	±	0.0009
bta-miR-128	0.0091	±	0.0079	0.0087	±	0.0104	0.0016	±	0.0011
bta-miR-1281	0.0342	±	0.0186	0.0377	±	0.0167	2.7083	±	0.2929
bta-miR-1287	0.0022	±	0.0012	0.0008	±	0.0005	0.0622	±	0.0610
bta-miR-129	0.0025	±	0.0016	0.0040	±	0.0027	0.0253	±	0.0076
bta-miR-129-5p	0.0023	±	0.0010	0.0035	±	0.0029	0.0136	±	0.0014
bta-miR-130a	0.0068	±	0.0050	0.0036	±	0.0009	0.0135	±	0.0029
bta-miR-130b	0.0101	±	0.0027	0.0079	±	0.0030	0.0222	±	0.0077
bta-miR-132	0.1163	±	0.0001	0.1441	±	0.0320	0.1328	±	0.2099
bta-miR-133a	0.0012	±	0.0008	0.0013	±	0.0013	0.1335	±	0.1083
bta-miR-134	0.0033	±	0.0003	0.0024	±	0.0012	0.1272	±	0.2080
bta-miR-135a	0.0134	±	0.0061	0.0220	±	0.0126	.	±	.
bta-miR-135b	0.0062	±	0.0045	0.0147	±	0.0144	0.0003	±	0.0000

bta-miR-136	0.0030	±	0.0029	0.0005	±	0.0000	.	±	.
bta-miR-138	0.0119	±	0.0042	0.0090	±	0.0041	0.2403	±	0.2825
bta-miR-139	0.0093	±	0.0071	0.0043	±	0.0031	0.0222	±	0.0208
bta-miR-140	0.0084	±	0.0109	0.0072	±	0.0100	0.0238	±	0.0370
bta-miR-141	0.0048	±	0.0065	0.0065	±	0.0067	0.0392	±	0.0361
bta-miR-143	0.0697	±	0.0383	0.0843	±	0.0201	0.0253	±	0.0310
bta-miR-148b	0.0292	±	0.0384	0.0332	±	0.0408	0.0658	±	0.0205
bta-miR-149-3p	0.5544	±	0.1471	0.5893	±	0.4360	0.3350	±	0.0541
bta-miR-150	0.0068	±	0.0009	0.0063	±	0.0059	0.0364	±	0.0450
bta-miR-151-3p	0.0404	±	0.0098	0.0342	±	0.0054	0.0288	±	0.0273
bta-miR-151-5p	0.2233	±	0.1935	0.3127	±	0.2565	0.0280	±	0.0040
bta-miR-152	0.0021	±	0.0012	0.0027	±	0.0003	0.0572	±	0.0963
bta-miR-153	0.0075	±	0.0101	0.0091	±	0.0098	0.0094	±	0.0068
bta-miR-154a	0.0008	±	0.0008	0.0008	±	0.0001	0.0605	±	0.0887
bta-miR-154b	0.0355	±	0.0122	0.0224	±	0.0081	0.2430	±	0.3430
bta-miR-181a	0.0010	±	0.0008	0.0026	±	0.0023	0.0006	±	0.0002
bta-miR-181b	0.0012	±	0.0005	0.0022	±	0.0027	0.0078	±	0.0023
bta-miR-181d	0.0010	±	0.0010	0.0013	±	0.0015	0.0344	±	0.0446
bta-miR-184	0.0016	±	0.0010	0.0008	±	0.0008	.	±	.
bta-miR-185	0.0008	±	0.0004	0.0029	±	0.0031	0.0050	±	0.0022
bta-miR-186	0.0385	±	0.0329	0.0441	±	0.0437	0.0322	±	0.0373
bta-miR-187	0.0095	±	0.0028	0.0061	±	0.0017	0.0954	±	0.0535
bta-miR-188	0.0128	±	0.0056	0.0122	±	0.0043	0.0277	±	0.0103
bta-miR-190a	0.0010	±	0.0001	0.0014	±	0.0006	0.0080	±	0.0085
bta-miR-190b	0.0326	±	0.0126	0.0361	±	0.0072	0.0058	±	0.0023
bta-miR-191	0.0526	±	0.0461	0.0566	±	0.0330	0.0654	±	0.0763
bta-miR-192	0.0160	±	0.0122	0.0180	±	0.0156	0.0069	±	0.0010
bta-miR-193a-3p	0.0006	±	0.0004	0.0008	±	0.0004	0.0029	±	0.0002
bta-miR-193a-5p	0.0027	±	0.0020	0.0026	±	0.0028	0.0102	±	0.0014
bta-miR-193b	0.0004	±	0.0001	0.0004	±	0.0004	0.0020	±	0.0020
bta-miR-194	0.0202	±	0.0093	0.0463	±	0.0416	0.0073	±	0.0051

bta-miR-196a	0.0067	±	0.0020	0.0099	±	0.0053	0.0633	±	0.0871
bta-miR-196b	0.0040	±	0.0046	0.0033	±	0.0013	0.0054	±	0.0083
bta-miR-199a-3p	0.0010	±	0.0003	0.0015	±	0.0010	.	±	.
bta-miR-199c	0.0023	±	0.0006	0.0037	±	0.0022	0.0487	±	0.0740
bta-miR-200a	0.0550	±	0.0092	0.0607	±	0.0690	.	±	.
bta-miR-200b	0.2199	±	0.1115	0.3415	±	0.0697	0.0109	±	0.0144
bta-miR-200c	0.0097	±	0.0051	0.0141	±	0.0025	0.0119	±	0.0172
bta-miR-204	0.2858	±	0.2761	0.3263	±	0.2577	0.0809	±	0.1048
bta-miR-205	0.0018	±	0.0002	0.0006	±	0.0002	0.0512	±	0.0420
bta-miR-206	0.0021	±	0.0016	0.0014	±	0.0008	0.0838	±	0.1251
bta-miR-210	0.0135	±	0.0061	0.0119	±	0.0082	0.0353	±	0.0061
bta-miR-211	0.2784	±	0.2428	0.2881	±	0.2613	0.7258	±	1.1919
bta-miR-214	0.0033	±	0.0005	0.0059	±	0.0020	0.0202	±	0.0012
bta-miR-215	0.0084	±	0.0054	0.0107	±	0.0087	0.0050	±	0.0030
bta-miR-216a	0.0017	±	0.0018	0.0047	±	0.0024	0.0256	±	0.0060
bta-miR-216b	0.0100	±	0.0045	0.0166	±	0.0022	0.0016	±	0.0014
bta-miR-217	0.0003	±	0.0001	0.0004	±	0.0003	.	±	.
bta-miR-219-3p	0.0015	±	0.0014	0.0008	±	0.0004	0.0245	±	0.0300
bta-miR-222	0.0009	±	0.0005	0.0009	±	0.0007	0.0355	±	0.0280
bta-miR-296-3p	0.0088	±	0.0074	0.0075	±	0.0020	0.1602	±	0.1695
bta-miR-296-5p	0.0358	±	0.0255	0.0362	±	0.0210	1.4022	±	1.0605
bta-miR-302c	0.0022	±	0.0017	0.0006	±	0.0002	0.0214	±	0.0358
bta-miR-320a	0.0427	±	0.0173	0.0325	±	0.0064	0.0776	±	0.0262
bta-miR-320b	0.0052	±	0.0015	0.0058	±	0.0015	0.0178	±	0.0134
bta-miR-323	133.069	±	68.9476	101.8617	±	48.3551	535.5672	±	82.9623
bta-miR-324	0.0065	±	0.0045	0.0062	±	0.0026	0.0116	±	0.0124
bta-miR-326	0.0124	±	0.0061	0.0154	±	0.0118	0.0669	±	0.0070
bta-miR-328	0.0076	±	0.0037	0.0088	±	0.0068	0.0182	±	0.0136
bta-miR-330	0.0050	±	0.0053	0.0040	±	0.0030	0.0194	±	0.0080
bta-miR-331-5p	0.0026	±	0.0014	0.0020	±	0.0008	0.0272	±	0.0165
bta-miR-335	0.0520	±	0.0597	0.0476	±	0.0568	.	±	.

bta-miR-338	0.0017	±	0.0000	0.0055	±	0.0049	0.0092	±	0.0055
bta-miR-339a	0.0082	±	0.0030	0.0142	±	0.0039	0.0796	±	0.0611
bta-miR-339b	0.0110	±	0.0035	0.0135	±	0.0120	0.2950	±	0.3178
bta-miR-345-3p	0.0021	±	0.0015	0.0024	±	0.0010	0.0209	±	0.0037
bta-miR-345-5p	0.0028	±	0.0017	0.0042	±	0.0020	0.0200	±	0.0108
bta-miR-346	0.0240	±	0.0121	0.0217	±	0.0147	0.3168	±	0.0560
bta-miR-363	0.0024	±	0.0024	0.0033	±	0.0034	0.0140	±	0.0041
bta-miR-365-5p	0.0034	±	0.0002	0.0057	±	0.0012	0.0174	±	0.0041
bta-miR-369-3p	0.0034	±	0.0024	0.0020	±	0.0014	.	±	.
bta-miR-370	0.0033	±	0.0027	0.0031	±	0.0010	0.0255	±	0.0075
bta-miR-374a	0.0017	±	0.0012	0.0021	±	0.0001	.	±	.
bta-miR-374b	0.0022	±	0.0009	0.0041	±	0.0008	.	±	.
bta-miR-375	0.2240	±	0.0873	0.2036	±	0.0566	0.0753	±	0.1085
bta-miR-378	0.0024	±	0.0022	0.0025	±	0.0014	0.1925	±	0.1854
bta-miR-378b	0.0016	±	0.0008	0.0015	±	0.0009	0.1624	±	0.1365
bta-miR-378d	0.0023	±	0.0025	0.0013	±	0.0003	0.2972	±	0.2940
bta-miR-380-5p	0.0214	±	0.0056	0.0099	±	0.0087	0.2800	±	0.2844
bta-miR-381	0.0043	±	0.0031	0.0023	±	0.0028	0.0056	±	0.0016
bta-miR-382	0.0304	±	0.0095	0.0207	±	0.0184	0.1174	±	0.0504
bta-miR-383	0.0022	±	0.0006	0.0014	±	0.0008	0.0198	±	0.0199
bta-miR-409a	0.0045	±	0.0072	0.0006	±	0.0002	.	±	.
bta-miR-410	0.0004	±	0.0001	0.0004	±	0.0001	0.0049	±	0.0044
bta-miR-411a	0.0036	±	0.0018	0.0022	±	0.0017	0.0403	±	0.0310
bta-miR-421	0.0070	±	0.0064	0.0067	±	0.0021	0.1600	±	0.1417
bta-miR-423-3p	0.0029	±	0.0023	0.0036	±	0.0030	0.0216	±	0.0044
bta-miR-423-5p	0.0100	±	0.0034	0.0126	±	0.0044	0.0031	±	0.0010
bta-miR-425-5p	1.1448	±	0.3236	1.2706	±	0.2768	0.0051	±	0.0006
bta-miR-429	0.0144	±	0.0073	0.0109	±	0.0036	0.0388	±	0.0065
bta-miR-431	0.0026	±	0.0014	0.0027	±	0.0019	0.0149	±	0.0038
bta-miR-432	0.0029	±	0.0015	0.0008	±	0.0008	0.0796	±	0.0897
bta-miR-433	0.0099	±	0.0026	0.0081	±	0.0053	0.1321	±	0.0795

bta-miR-449a	0.3221	±	0.3608	0.2782	±	0.3492	0.0034	±	0.0012
bta-miR-449b	0.0206	±	0.0301	0.0301	±	0.0446	0.0101	±	0.0078
bta-miR-449d	0.0132	±	0.0058	0.0108	±	0.0074	0.0335	±	0.0128
bta-miR-452	0.0013	±	0.0012	0.0002	±	0.0000	0.0328	±	0.0395
bta-miR-453	0.0069	±	0.0001	0.0039	±	0.0005	0.0046	±	0.0035
bta-miR-484	0.0007	±	0.0007	0.0012	±	0.0010	0.0332	±	0.0101
bta-miR-485	0.0005	±	0.0002	0.0004	±	0.0004	0.0082	±	0.0103
bta-miR-486	0.0694	±	0.0216	0.0555	±	0.0103	0.1009	±	0.0403
bta-miR-487b	0.0012	±	0.0008	0.0006	±	0.0006	0.0027	±	0.0023
bta-miR-489	0.0016	±	0.0011	0.0010	±	0.0004	0.0482	±	0.0301
bta-miR-490	0.0039	±	0.0007	0.0047	±	0.0028	0.0339	±	0.0150
bta-miR-491	0.0012	±	0.0007	0.0029	±	0.0037	0.0221	±	0.0168
bta-miR-493	0.0106	±	0.0053	0.0105	±	0.0037	0.0527	±	0.0298
bta-miR-494	1.3068	±	0.7157	2.2816	±	1.0446	10.5780	±	3.0809
bta-miR-497	0.0097	±	0.0084	0.0113	±	0.0112	0.0055	±	0.0041
bta-miR-500	0.0011	±	0.0002	0.0008	±	0.0001	0.1472	±	0.0597
bta-miR-503-3p	0.0199	±	0.0085	0.0115	±	0.0028	0.2895	±	0.1169
bta-miR-504	0.0027	±	0.0024	0.0033	±	0.0008	0.0994	±	0.0753
bta-miR-505	0.0033	±	0.0001	0.0009	±	0.0003	0.0320	±	0.0253
bta-miR-532	0.0139	±	0.0207	0.0002	±	0.0002	0.0095	±	0.0027
bta-miR-541	0.0150	±	0.0027	0.0141	±	0.0050	0.1668	±	0.0058
bta-miR-542-5p	0.0110	±	0.0059	0.0034	±	0.0017	0.0005	±	0.0002
bta-miR-545-5p	0.0024	±	0.0012	0.0007	±	0.0003	.	±	.
bta-miR-574	0.1758	±	0.0904	0.1467	±	0.0745	1.8761	±	0.5947
bta-miR-584	0.0031	±	0.0017	0.0031	±	0.0017	0.0298	±	0.0138
bta-miR-631	29.1166	±	16.7781	13.9559	±	3.3240	86.5707	±	17.6398
bta-miR-654	0.0025	±	0.0002	0.0026	±	0.0037	0.0218	±	0.0137
bta-miR-656	0.0030	±	0.0006	0.0059	±	0.0075	0.0232	±	0.0123
bta-miR-658	0.0019	±	0.0018	0.0011	±	0.0007	0.0024	±	0.0009
bta-miR-660	0.0076	±	0.0010	0.0089	±	0.0055	0.0347	±	0.0334
bta-miR-664a	0.0113	±	0.0042	0.0186	±	0.0155	0.0312	±	0.0099

bta-miR-664b	0.0277	±	0.0202	0.0274	±	0.0151	0.0520	±	0.0315
bta-miR-665	0.0852	±	0.0075	0.0491	±	0.0148	1.8669	±	0.5909
bta-miR-669	0.0136	±	0.0064	0.0152	±	0.0086	0.2193	±	0.0717
bta-miR-677	0.0036	±	0.0029	0.0011	±	0.0002	0.0120	±	0.0074
bta-miR-708	0.0009	±	0.0002	0.0007	±	0.0004	0.0137	±	0.0096
bta-miR-744	0.0005	±	0.0001	0.0011	±	0.0007	0.0034	±	0.0011
bta-miR-760-3p	0.0084	±	0.0033	0.0064	±	0.0049	0.0605	±	0.0178
bta-miR-760-5p	0.1440	±	0.1134	0.2701	±	0.2594	0.2879	±	0.0473
bta-miR-761	0.0017	±	0.0005	0.0017	±	0.0015	0.0019	±	0.0020
bta-miR-763	0.0047	±	0.0032	0.0170	±	0.0118	0.0537	±	0.0226
bta-miR-764	0.0296	±	0.0388	0.0177	±	0.0243	0.0095	±	0.0053
bta-miR-767	0.0068	±	0.0045	0.0056	±	0.0016	0.0156	±	0.0003
bta-miR-769	0.0010	±	0.0003	0.0007	±	0.0006	0.0265	±	0.0138
bta-miR-873	0.0009	±	0.0012	0.0003	±	0.0003	0.0258	±	0.0200
bta-miR-877	0.0348	±	0.0057	0.0213	±	0.0024	0.1232	±	0.0367
bta-miR-885	0.0025	±	0.0026	0.0031	±	0.0020	0.0446	±	0.0129
bta-miR-935	0.0072	±	0.0025	0.0080	±	0.0044	0.2452	±	0.0586
bta-miR-940	0.9009	±	0.3382	0.7745	±	0.1527	32.4275	±	12.2599
bta-miR-1224	3.2663	±	1.3077	3.1231	±	0.5453	2.3409	±	0.6572
bta-miR-1246	0.9191	±	0.4005	1.2088	±	0.4008	12.6860	±	4.3288
bta-miR-1247-3p	0.0213	±	0.0070	0.0135	±	0.0013	0.2544	±	0.1685
bta-miR-1247-5p	0.3421	±	0.1552	0.3060	±	0.1354	0.4988	±	0.2081
bta-miR-1249	0.0018	±	0.0013	0.0014	±	0.0012	0.0401	±	0.0039
bta-miR-1291	0.0005	±	0.0003	0.0014	±	0.0008	0.0118	±	0.0070
bta-miR-1296	0.0181	±	0.0065	0.0193	±	0.0015	.	±	.
bta-miR-1306	0.0258	±	0.0200	0.0246	±	0.0120	0.3889	±	0.3027
bta-miR-1307	0.0654	±	0.0223	0.1022	±	0.0375	0.7877	±	0.2135
bta-miR-1343-3p	0.0224	±	0.0115	0.0252	±	0.0090	0.3794	±	0.0953
bta-miR-1343-5p	1.2755	±	0.6539	0.9952	±	0.1259	0.4247	±	0.1212
bta-miR-1388-3p	0.0090	±	0.0056	0.0060	±	0.0019	0.1593	±	0.0131
bta-miR-4523	0.0113	±	0.0053	0.0088	±	0.0035	0.0115	±	0.0047

Hm/Ms/RtT1snRNA	0.9175	±	0.2472	1.6149	±	0.5084	53.6440	±	17.0054
RNT43 snoRNA	0.0041	±	0.0025	0.0045	±	0.0006	0.1462	±	0.0139
Exclusively detected in HF group of sperm cells									
bta-miR-27a-5p	0.0010	±	0.0005	.	±	.	0.0035	±	0.0021
bta-miR-31	0.0022	±	0.0003	.	±	.	0.0146	±	0.0072
bta-miR-32	0.0006	±	0.0005	.	±	.	.	±	.
bta-miR-133b	0.0004	±	0.0003	.	±	.	0.0074	±	0.0071
bta-miR-142-5p	0.0009	±	0.0009	.	±	.	.	±	.
bta-miR-145	0.0008	±	0.0001	.	±	.	0.1698	±	0.1044
bta-miR-146a	0.0142	±	0.0129	.	±	.	0.0051	±	0.0037
bta-miR-147	0.0028	±	0.0011	.	±	.	0.0008	±	0.0009
bta-miR-149-5p	0.0011	±	0.0008	.	±	.	1.5298	±	1.5311
bta-miR-154c	0.0012	±	0.0005	.	±	.	0.0114	±	0.0187
bta-miR-212	0.0017	±	0.0000	.	±	.	0.0071	±	0.0088
bta-miR-219	0.0027	±	0.0011	.	±	.	0.0703	±	0.0348
bta-miR-301b	0.0002	±	0.0002	.	±	.	.	±	.
bta-miR-302b	0.0007	±	0.0004	.	±	.	.	±	.
bta-miR-331-3p	0.0009	±	0.0001	.	±	.	.	±	.
bta-miR-380-3p	0.0013	±	0.0001	.	±	.	0.0206	±	0.0159
bta-miR-411b	0.0003	±	0.0002	.	±	.	0.0041	±	0.0028
bta-miR-425-3p	0.0827	±	0.0364	.	±	.	14.1388	±	5.8179
bta-miR-495	0.0022	±	0.0009	.	±	.	.	±	.
bta-miR-502b	0.0023	±	0.0001	.	±	.	0.0378	±	0.0310
bta-miR-545-3p	0.0012	±	0.0004	.	±	.	.	±	.
bta-miR-655	0.0004	±	0.0003	.	±	.	0.0054	±	0.0044
bta-miR-874	0.0227	±	0.0104	.	±	.	0.0836	±	0.0124
bta-miR-1225-3p	0.3219	±	0.1637	.	±	.	26.8475	±	0.9828
Exclusively detected in LF group of sperm cells									
bta-let-7b	.	±	.	0.0878	±	0.1248	0.0021	±	0.0016
bta-miR-17-3p	.	±	.	0.0016	±	0.0012	0.0060	±	0.0038
bta-miR-21-3p	.	±	.	0.0001	±	0.0000	0.0051	±	0.0020

bta-miR-107	.	±	.	0.0006	±	0.0001	0.1329	±	0.1836
bta-miR-129-3p	.	±	.	0.0005	±	0.0002	0.0038	±	0.0020
bta-miR-137	.	±	.	0.0027	±	0.0015	0.0011	±	0.0002
bta-miR-146b	.	±	.	0.0066	±	0.0039	0.0080	±	0.0064
bta-miR-148a	.	±	.	0.0271	±	0.0317	0.1130	±	0.0373
bta-miR-181c	.	±	.	0.0017	±	0.0006	.	±	.
bta-miR-195	.	±	.	0.7969	±	0.7520	0.0367	±	0.0193
bta-miR-301a	.	±	.	0.0003	±	0.0003	.	±	.
bta-miR-302a	.	±	.	0.0017	±	0.0020	.	±	.
bta-miR-361	.	±	.	0.0002	±	0.0001	0.0065	±	0.0043
bta-miR-362-3p	.	±	.	0.0013	±	0.0012	0.0144	±	0.0157
bta-miR-362-5p	.	±	.	0.0003	±	0.0003	0.0025	±	0.0010
bta-miR-371	.	±	.	0.0005	±	0.0005	0.0018	±	0.0013
bta-miR-376a	.	±	.	0.0003	±	0.0001	.	±	.
bta-miR-377	.	±	.	0.0010	±	0.0006	0.0007	±	0.0005
bta-miR-378c	.	±	.	0.0013	±	0.0009	0.0020	±	0.0020
bta-miR-379	.	±	.	0.0007	±	0.0004	0.0023	±	0.0011
bta-miR-424-3p	.	±	.	0.0007	±	0.0003	0.0135	±	0.0046
bta-miR-424-5p	.	±	.	0.0010	±	0.0012	0.0239	±	0.0151
bta-miR-448	.	±	.	0.0001	±	0.0000	0.0028	±	0.0008
bta-miR-455-5p	.	±	.	0.0011	±	0.0013	0.0716	±	0.0709
bta-miR-483	.	±	.	0.0010	±	0.0008	0.0061	±	0.0033
bta-miR-488	.	±	.	0.0008	±	0.0004	0.1997	±	0.1782
bta-miR-499	.	±	.	0.0007	±	0.0005	.	±	.
bta-miR-502a	.	±	.	0.0005	±	0.0003	0.0033	±	0.0031
bta-miR-503-5p	.	±	.	0.0004	±	0.0002	0.0414	±	0.0183
bta-miR-543	.	±	.	0.0021	±	0.0001	0.0046	±	0.0038
bta-miR-670	.	±	.	0.0012	±	0.0006	.	±	.
bta-miR-671	.	±	.	0.0005	±	0.0000	0.0005	±	0.0002
Not detected in either group (HF and LF) of sperm cells									
bta-miR-1	.	±	.	.	±	.	.	±	.

bta-miR-9-3p	.	±	.	.	±	.	.	±	.
bta-miR-22-3p	.	±	.	.	±	.	.	±	.
bta-miR-23b-5p	.	±	.	.	±	.	0.0016	±	0.0009
bta-miR-24	.	±	.	.	±	.	.	±	.
bta-miR-26c	.	±	.	.	±	.	.	±	.
bta-miR-30b-3p	.	±	.	.	±	.	.	±	.
bta-miR-33a	.	±	.	.	±	.	0.0021	±	0.0002
bta-miR-96	.	±	.	.	±	.	.	±	.
bta-miR-98	.	±	.	.	±	.	.	±	.
bta-miR-99a-3p	.	±	.	.	±	.	0.0049	±	0.0021
bta-miR-133c	.	±	.	.	±	.	0.0857	±	0.1468
bta-miR-142-3p	.	±	.	.	±	.	.	±	.
bta-miR-144	.	±	.	.	±	.	.	±	.
bta-miR-155	.	±	.	.	±	.	0.0170	±	0.0238
bta-miR-182	.	±	.	.	±	.	0.0238	±	0.0328
bta-miR-183	.	±	.	.	±	.	0.0519	±	0.0370
bta-miR-193a	.	±	.	.	±	.	0.0006	±	0.0004
bta-miR-197	.	±	.	.	±	.	0.4294	±	0.5134
bta-miR-199a-5p	.	±	.	.	±	.	0.0009	±	0.0007
bta-miR-199b	.	±	.	.	±	.	0.0319	±	0.0304
bta-miR-202	.	±	.	.	±	.	0.0033	±	0.0014
bta-miR-208a	.	±	.	.	±	.	.	±	.
bta-miR-208b	.	±	.	.	±	.	.	±	.
bta-miR-219-5p	.	±	.	.	±	.	.	±	.
bta-miR-221	.	±	.	.	±	.	0.0199	±	0.0074
bta-miR-223	.	±	.	.	±	.	0.0089	±	0.0064
bta-miR-224	.	±	.	.	±	.	.	±	.
bta-miR-299	.	±	.	.	±	.	.	±	.
bta-miR-302d	.	±	.	.	±	.	0.0025	±	0.0006
bta-miR-329a	.	±	.	.	±	.	.	±	.
bta-miR-329b	.	±	.	.	±	.	.	±	.

bta-miR-340	.	±	.	.	±	.	0.1876	±	0.2809
bta-miR-342	.	±	.	.	±	.	0.0220	±	0.0215
bta-miR-365-3p	.	±	.	.	±	.	.	±	.
bta-miR-367	.	±	.	.	±	.	.	±	.
bta-miR-369-5p	.	±	.	.	±	.	0.0388	±	0.0515
bta-miR-376b	.	±	.	.	±	.	.	±	.
bta-miR-376c	.	±	.	.	±	.	.	±	.
bta-miR-376d	.	±	.	.	±	.	.	±	.
bta-miR-376e	.	±	.	.	±	.	.	±	.
bta-miR-409b	.	±	.	.	±	.	0.0360	±	0.0278
bta-miR-411c-3p	.	±	.	.	±	.	.	±	.
bta-miR-411c-5p	.	±	.	.	±	.	.	±	.
bta-miR-412	.	±	.	.	±	.	0.0023	±	0.0015
bta-miR-449c	.	±	.	.	±	.	0.0181	±	0.0242
bta-miR-450a	.	±	.	.	±	.	.	±	.
bta-miR-450b	.	±	.	.	±	.	.	±	.
bta-miR-451	.	±	.	.	±	.	.	±	.
bta-miR-454	.	±	.	.	±	.	0.0457	±	0.0539
bta-miR-455-3p	.	±	.	.	±	.	0.0085	±	0.0061
bta-miR-487a	.	±	.	.	±	.	0.0021	±	0.0008
bta-miR-496	.	±	.	.	±	.	0.0978	±	0.0761
bta-miR-539	.	±	.	.	±	.	0.0014	±	0.0016
bta-miR-544a	.	±	.	.	±	.	0.0100	±	0.0129
bta-miR-544b	.	±	.	.	±	.	0.0132	±	0.0056
bta-miR-551a	.	±	.	.	±	.	0.0043	±	0.0038
bta-miR-551b	.	±	.	.	±	.	.	±	.
bta-miR-562	.	±	.	.	±	.	0.0042	±	0.0010
bta-miR-568	.	±	.	.	±	.	0.0020	±	0.0017
bta-miR-582	.	±	.	.	±	.	0.1427	±	0.0503
bta-miR-592	.	±	.	.	±	.	0.0020	±	0.0002
bta-miR-599	.	±	.	.	±	.	.	±	.

bta-miR-615	.	±	.	.	±	.	40790.7953	±	2884.1670
bta-miR-628	.	±	.	.	±	.	0.0363	±	0.0567
bta-miR-652	.	±	.	.	±	.	0.0110	±	0.0048
bta-miR-653	.	±	.	.	±	.	.	±	.
bta-miR-758	.	±	.	.	±	.	0.1031	±	0.0769
bta-miR-759	.	±	.	.	±	.	.	±	.
bta-miR-875	.	±	.	.	±	.	0.0028	±	0.0014
bta-miR-876	.	±	.	.	±	.	0.4186	±	0.3493
bta-miR-1179	.	±	.	.	±	.	0.0090	±	0.0124
bta-miR-1185	.	±	.	.	±	.	.	±	.
bta-miR-1193	.	±	.	.	±	.	0.0185	±	0.0213
bta-miR-1197	.	±	.	.	±	.	.	±	.
bta-miR-1248	.	±	.	.	±	.	0.0205	±	0.0186
bta-miR-1271	.	±	.	.	±	.	.	±	.
bta-miR-1277	.	±	.	.	±	.	.	±	.
bta-miR-1282	.	±	.	.	±	.	0.0031	±	0.0026
bta-miR-1284	.	±	.	.	±	.	0.0202	±	0.0191
bta-miR-1298	.	±	.	.	±	.	0.0032	±	0.0022
bta-miR-1301	.	±	.	.	±	.	0.0099	±	0.0094
bta-miR-3064	.	±	.	.	±	.	0.0025	±	0.0006

Supplementary Table S6. Mean and SEM of relative levels of the nine miRNAs investigated in zygotes from high fertility (HF), low fertility (LF) and parthenogenesis (PA) groups as well as mature oocytes (OO). [1]High fertility; [2]Low fertility; [3]Parthenogenetic; [4]Mature oocytes. SEM: standard error of the mean.

Zygotes	HF[1] (n=9)	LF[2] (n=9)	PA[3] (n=9)	OO[4] (n=3)
bta-miR-33b	0.001±0.0001	0.002±0.0009	0.001±0.0002	0.001±0.0004
bta-miR-126-5p	0.0008±0.0001	0.002±0.001	0.001±0.0006	0.0004±0.0001
bta-miR-205	0.027±0.007	0.06±0.03	0.02±0.003	0.02±0.01
bta-miR-216b	0.0006±0.0001[b]	0.002±0.00003[a]	0.001±0.0003[ab]	0.0006±0.0003[b]
bta-miR-339a	0.020±0.002	0.01±0.002	0.01±0.003	0.01±0.003
bta-miR-500	0.001±0.0004	0.005±0.005	0.0007±0.0001	0.001±0.0005
bta-miR-505	0.013±0.002	0.02±0.01	0.01±0.001	0.01±0.003
bta-miR-532	0.001±0.0004	0.006±0.005	0.002±0.0006	0.0009±0.0002
bta-miR-542-5p	0.0009±0.0003	0.006±0.006	0.0005±0.0002	0.0006±0.0003

Supplementary Table S7. Mean and SEM of relative levels of the nine miRNAs investigated in two-cell embryos from high fertility (HF), low fertility (LF) and parthenogenesis (PA) groups as well as mature oocytes (OO). [1]High fertility; [2]Low fertility; [3]Parthenogenetic; [4]Mature oocytes. SEM: standard error of the mean.

Two-cell embryos	HF[1] (n=9)	LF[2] (n=9)	PA[3] (n=9)	OO[4] (n=3)
bta-miR-33b	0.001±0.0003	0.002±0.005	0.001±0.0004	0.001±0.0004
bta-miR-126-5p	0.05±0.02[a]	0.07±0.04[a]	0.02±0.01[a]	0.004±0.0001[b]
bta-miR-205	0.04±0.01	0.05±0.01	0.05±0.01	0.02±0.01
bta-miR-216b	0.002±0.0005	0.001±0.0002	0.001±0.0005	0.0006±0.0003
bta-miR-339a	0.12±0.02[a]	0.12±0.03[a]	0.08±0.01[a]	0.01±0.003[b]
bta-miR-500	0.13±0.06	0.10±0.05	0.15±0.08	0.001±0.0005
bta-miR-505	0.05±0.01	0.06±0.02	0.05±0.01	0.01±0.003
bta-miR-532	0.02±0.01[a]	0.02±0.01[a]	0.01±0.007[a]	0.0009±0.0002[b]
bta-miR-542-5p	0.002±0.0005	0.003±0.0006	0.001±0.0005	0.0006±0.0003

Supplementary Table S8. Pre-implantation development rates of bovine embryos produced *in vitro* with IVF of 8 hours for kinetics evaluation. Two-cell (evaluated 28 to 30 hpi) and four-cell (evaluated 40 to 42 hpi) rates were evaluated in embryos produced *in vitro* using high and low fertility semen samples. [*a,b] Different letters on the same column indicate tendency to statistical difference (P=0.07) between groups. [1]High fertility; [2]Low fertility. Hpi: hours post insemination. Mean and SEM are presented as percentages. SEM: standard error of the mean.

Fertility Groups	Two-cell embryos			Four-cell embryos		
	Oocytes	N	Mean±SEM	Oocytes	N	Mean±SEM
HF[1]	328	115	35.1±6.8*[a]	489	124	25.4±4.2
LF[2]	365	104	28.5±6.7*[b]	482	129	26.8±3.2

Supplementary Table S9. Classification of morphologically abnormal spermatozoa with regard to major and minor defects adapted from Blom [46].

Major Defects	Minor Defects
Acrosome defects	Head defects:
Proximal Droplet	Narrow
Head defects:	Giant, short, broad, and small normal
Underdeveloped	Free normal
Tail curled in the head	Abaxial, Retroaxial, Oblique implantation
Free pathological heads	Simple bent tail
Narrow at base	Distal droplet
Pear-shaped defect	
Small abnormal heads	
Abnormal contour	
Pouch formation	
Double forms	
Midpiece defects:	
Corkscrew defect, Pseudodroplet, Tail stump, Broken neck, Swelling and others	
Tail defects:	
Strongly coiled or folded tail	
Coiled with distal droplet attached	

Supplementary Table S10. Set up for bovine sperm kinetic evaluation on Sperm Class Analyzer (Microptic, Barcelona, Spain).

**Characteristic**	**Adjusted to**
Particle area	15 to 70 μm^2
Fast sperm	VAP (path velocity) > 40 μm/s
Progressive motility	STR (straightness) > 70%
Circular movement	LIN (linearity) < 50%
VAP points	5
Number of images	30
Images per second	50

Supplementary Table S11. Panel of 380 miRNAs investigated by qPCR in sperm cells.

#	miRNA	#	miRNA	#	miRNA	#	miRNA	#	miRNA
1	bta-let-7a-3p	77	bta-miR-142-5p	153	bta-miR-216a	229	bta-miR-361	305	bta-miR-496
2	bta-let-7a-5p	78	bta-miR-143	154	bta-miR-216b	230	bta-miR-362-3p	306	bta-miR-497
3	bta-let-7b	79	bta-miR-144	155	bta-miR-217	231	bta-miR-362-5p	307	bta-miR-499
4	bta-let-7c	80	bta-miR-145	156	bta-miR-218	232	bta-miR-363	308	bta-miR-500
5	bta-let-7d	81	bta-miR-146a	157	bta-miR-219	233	bta-miR-365-3p	309	bta-miR-502a
6	bta-let-7e	82	bta-miR-146b	158	bta-miR-219-3p	234	bta-miR-365-5p	310	bta-miR-502b
7	bta-let-7f	83	bta-miR-147	159	bta-miR-219-5p	235	bta-miR-367	311	bta-miR-503-3p
8	bta-let-7g	84	bta-miR-148a	160	bta-miR-221	236	bta-miR-369-3p	312	bta-miR-503-5p
9	bta-let-7i	85	bta-miR-148b	161	bta-miR-222	237	bta-miR-369-5p	313	bta-miR-504
10	bta-miR-1	86	bta-miR-149-3p	162	bta-miR-223	238	bta-miR-370	314	bta-miR-505
11	bta-miR-100	87	bta-miR-149-5p	163	bta-miR-22-3p	239	bta-miR-371	315	bta-miR-532
12	bta-miR-101	88	bta-miR-150	164	bta-miR-224	240	bta-miR-374a	316	bta-miR-539
13	bta-miR-103	89	bta-miR-151-3p	165	bta-miR-22-5p	241	bta-miR-374b	317	bta-miR-541
14	bta-miR-105a	90	bta-miR-151-5p	166	bta-miR-23a	242	bta-miR-375	318	bta-miR-542-5p
15	bta-miR-105b	91	bta-miR-152	167	bta-miR-23b-3p	243	bta-miR-376a	319	bta-miR-543
16	bta-miR-106a	92	bta-miR-153	168	bta-miR-23b-5p	244	bta-miR-376b	320	bta-miR-544a
17	bta-miR-106b	93	bta-miR-154a	169	bta-miR-24	245	bta-miR-376c	321	bta-miR-544b

18	bta-miR-107	94	bta-miR-154b	170	bta-miR-24-3p	246	bta-miR-376d	322	bta-miR-545-3p
19	bta-miR-10a	95	bta-miR-154c	171	bta-miR-25	247	bta-miR-376e	323	bta-miR-545-5p
20	bta-miR-10b	96	bta-miR-155	172	bta-miR-26a	248	bta-miR-377	324	bta-miR-551a
21	bta-miR-1179	97	bta-miR-15a	173	bta-miR-26b	249	bta-miR-378	325	bta-miR-551b
22	bta-miR-1185	98	bta-miR-15b	174	bta-miR-26c	250	bta-miR-378b	326	bta-miR-562
23	bta-miR-1193	99	bta-miR-16a	175	bta-miR-27a-3p	251	bta-miR-378c	327	bta-miR-568
24	bta-miR-1197	100	bta-miR-16b	176	bta-miR-27a-5p	252	bta-miR-378d	328	bta-miR-574
25	bta-miR-122	101	bta-miR-17-3p	177	bta-miR-27b	253	bta-miR-379	329	bta-miR-582
26	bta-miR-122	102	bta-miR-17-5p	178	bta-miR-28	254	bta-miR-380-3p	330	bta-miR-584
27	bta-miR-1224	103	bta-miR-181a	179	bta-miR-296-3p	255	bta-miR-380-5p	331	bta-miR-592
28	bta-miR-1225-3p	104	bta-miR-181b	180	bta-miR-296-5p	256	bta-miR-381	332	bta-miR-599
29	bta-miR-1246	105	bta-miR-181c	181	bta-miR-299	257	bta-miR-382	333	bta-miR-615
30	bta-miR-1247-3p	106	bta-miR-181d	182	bta-miR-29a	258	bta-miR-383	334	bta-miR-628
31	bta-miR-1247-5p	107	bta-miR-182	183	bta-miR-29b	259	bta-miR-409a	335	bta-miR-631
32	bta-miR-1248	108	bta-miR-183	184	bta-miR-29c	260	bta-miR-409b	336	bta-miR-652
33	bta-miR-1249	109	bta-miR-184	185	bta-miR-29d-3p	261	bta-miR-410	337	bta-miR-653
34	bta-miR-124a	110	bta-miR-185	186	bta-miR-29d-5p	262	bta-miR-411a	338	bta-miR-654
35	bta-miR-124b	111	bta-miR-186	187	bta-miR-29e	263	bta-miR-411b	339	bta-miR-655
36	bta-miR-125a	112	bta-miR-187	188	bta-miR-301a	264	bta-miR-411c-3p	340	bta-miR-656

37	bta-miR-125b	113	bta-miR-188	189	bta-miR-301b	265	bta-miR-411c-5p	341	bta-miR-658
38	bta-miR-1260b	114	bta-miR-18a	190	bta-miR-302a	266	bta-miR-412	342	bta-miR-660
39	bta-miR-126-3p	115	bta-miR-18b	191	bta-miR-302b	267	bta-miR-421	343	bta-miR-664a
40	bta-miR-126-5p	116	bta-miR-190a	192	bta-miR-302c	268	bta-miR-423-3p	344	bta-miR-664b
41	bta-miR-127	117	bta-miR-190b	193	bta-miR-302d	269	bta-miR-423-5p	345	bta-miR-665
42	bta-miR-1271	118	bta-miR-191	194	bta-miR-3064	270	bta-miR-424-3p	346	bta-miR-669
43	bta-miR-1277	119	bta-miR-192	195	bta-miR-30a-5p	271	bta-miR-424-5p	347	bta-miR-670
44	bta-miR-128	120	bta-miR-193a	196	bta-miR-30b-3p	272	bta-miR-425-3p	348	bta-miR-671
45	bta-miR-1281	121	bta-miR-193a-3p	197	bta-miR-30b-5p	273	bta-miR-425-5p	349	bta-miR-677
46	bta-miR-1282	122	bta-miR-193a-5p	198	bta-miR-30c	274	bta-miR-429	350	bta-miR-7
47	bta-miR-1284	123	bta-miR-193b	199	bta-miR-30d	275	bta-miR-431	351	bta-miR-708
48	bta-miR-1287	124	bta-miR-194	200	bta-miR-30e-5p	276	bta-miR-432	352	bta-miR-744
49	bta-miR-129	125	bta-miR-195	201	bta-miR-30f	277	bta-miR-433	353	bta-miR-758
50	bta-miR-1291	126	bta-miR-196a	202	bta-miR-31	278	bta-miR-448	354	bta-miR-759
51	bta-miR-129-3p	127	bta-miR-196b	203	bta-miR-32	279	bta-miR-449a	355	bta-miR-760-3p
52	bta-miR-129-5p	128	bta-miR-197	204	bta-miR-320a	280	bta-miR-449b	356	bta-miR-760-5p
53	bta-miR-1296	129	bta-miR-199a-3p	205	bta-miR-320b	281	bta-miR-449c	357	bta-miR-761
54	bta-miR-1298	130	bta-miR-199a-5p	206	bta-miR-323	282	bta-miR-449d	358	bta-miR-763
55	bta-miR-1301	131	bta-miR-199b	207	bta-miR-324	283	bta-miR-450a	359	bta-miR-764

56	bta-miR-1306	132	bta-miR-199c	208	bta-miR-326	284	bta-miR-450b	360	bta-miR-767
57	bta-miR-1307	133	bta-miR-19a	209	bta-miR-328	285	bta-miR-451	361	bta-miR-769
58	bta-miR-130a	134	bta-miR-19b	210	bta-miR-329a	286	bta-miR-452	362	bta-miR-873
59	bta-miR-130b	135	bta-miR-200a	211	bta-miR-329b	287	bta-miR-4523	363	bta-miR-874
60	bta-miR-132	136	bta-miR-200b	212	bta-miR-330	288	bta-miR-453	364	bta-miR-875
61	bta-miR-133a	137	bta-miR-200c	213	bta-miR-331-3p	289	bta-miR-454	365	bta-miR-876
62	bta-miR-133b	138	bta-miR-202	214	bta-miR-331-5p	290	bta-miR-455-3p	366	bta-miR-877
63	bta-miR-133c	139	bta-miR-204	215	bta-miR-335	291	bta-miR-455-5p	367	bta-miR-885
64	bta-miR-134	140	bta-miR-205	216	bta-miR-338	292	bta-miR-483	368	bta-miR-92a
65	bta-miR-1343-3p	141	bta-miR-206	217	bta-miR-339a	293	bta-miR-484	369	bta-miR-92b
66	bta-miR-1343-5p	142	bta-miR-208a	218	bta-miR-339b	294	bta-miR-485	370	bta-miR-93
67	bta-miR-135a	143	bta-miR-208b	219	bta-miR-33a	295	bta-miR-486	371	bta-miR-935
68	bta-miR-135b	144	bta-miR-20a	220	bta-miR-33b	296	bta-miR-487a	372	bta-miR-9-3p
69	bta-miR-136	145	bta-miR-20b	221	bta-miR-340	297	bta-miR-487b	373	bta-miR-940
70	bta-miR-137	146	bta-miR-210	222	bta-miR-342	298	bta-miR-488	374	bta-miR-95
71	bta-miR-138	147	bta-miR-211	223	bta-miR-345-3p	299	bta-miR-489	375	bta-miR-9-5p
72	bta-miR-1388-3p	148	bta-miR-212	224	bta-miR-345-5p	300	bta-miR-490	376	bta-miR-96
73	bta-miR-139	149	bta-miR-21-3p	225	bta-miR-346	301	bta-miR-491	377	bta-miR-98
74	bta-miR-140	150	bta-miR-214	226	bta-miR-34a	302	bta-miR-493	378	bta-miR-99a-3p

75	bta-miR-141	151	bta-miR-215	227	bta-miR-34b	303	bta-miR-494	379	bta-miR-99a-5p
76	bta-miR-142-3p	152	bta-miR-21-5p	228	bta-miR-34c	304	bta-miR-495	380	bta-miR-99b

Supplementary Table S12. Sequences of forward and reverse miR-216b target gene primers used for qPCR.

Gene	Product size	Forward primer (5′-3′)	Reverse primer (5′-3′)	NCBI accession number
K-RAS	133	GCCAGTTAGCTTGGTAGGTGT	AACCAAGGCATGGCAACAAT	NM_001110001.2
BECN1	182	GCCTCTGAAACTGGACACGA	GGGGGATGAATCTGCGAGAG	NM_001033627.2
JUN	140	TGCAAACGTTTTGAGGCGAG	GGGCTTTAGTCCTCGGACAC	NM_001077827.1
β-ACTIN	158	TCGGTTGGATCGAGCATTCC	CCTTAGAGAGAAGCGGGGTG	NM_173979.3

Supplementary Figure S1. Preliminary results of polyspermic embryo induction. A. Zygote polyspermy rates by different sperm concentrations in *in vitro* fertilization drops. **B.** Number of oocytes and mean and SEM (%) of zygote polyspermy rates by different sperm concentrations in *in vitro* fertilization drops. Groups: Conc-1: 1×10^6 sperm/mL (n = 2); Conc-4: 4×10^6 sperm/mL (n = 2); Conc-8: 8×10^6 sperm/mL (n = 2); Conc-16: 16×10^6 sperm/mL (n = 2). For this experiment, two straws from one frozen-thawed commercial semen batch were used to perform two replicates of *in vitro* embryo production. Zygotes were collected 12 hours post insemination (hpi) to evaluate embryo polyspermy rate. SEM: standard error of the mean.

A

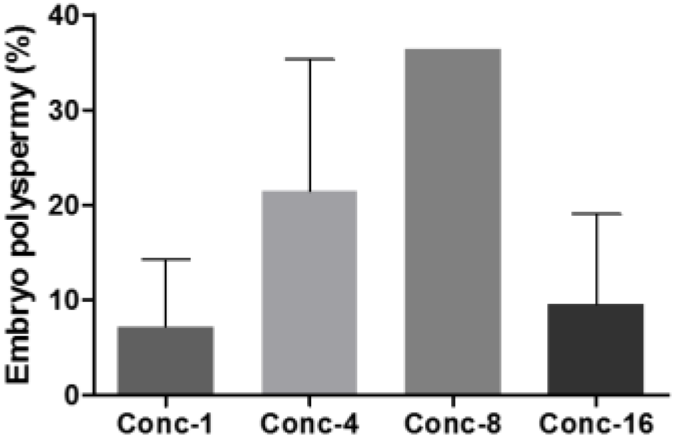

B

Groups	Number of oocytes	Total of polyspermy (%)
Conc-1 (1×10^6)	29	7.1±7.1
Conc-4 (4×10^6)	30	21.5±13.8
Conc-8 (8×10^6)	33	36.4±0
Conc-16 (16×10^6)	34	9.5±9.5
